# Shedding of CD16 disassembles the NK cell immune synapse and boosts serial engagement of target cells

**DOI:** 10.1083/jcb.201712085

**Published:** 2018-09-03

**Authors:** Katja Srpan, Ashley Ambrose, Alexandros Karampatzakis, Mezida Saeed, Adam N.R. Cartwright, Karolin Guldevall, Gabriela Dos Santos Cruz De Matos, Björn Önfelt, Daniel M. Davis

**Affiliations:** 1 The Lydia Becker Institute of Immunology and Inflammation, Manchester Collaborative Centre for Inflammation Research, University of Manchester, Manchester, UK; 2 Department of Applied Physics, Science for Life Laboratories, KTH Royal Institute of Technology, Solna, Sweden; 3 GlaxoSmithKline, Stevenage, UK; 4 Department of Microbiology, Tumor and Cell Biology, Karolinska Institutet, Stockholm, Sweden

## Abstract

Natural Killer (NK) cells can engage multiple virally infected or tumor cells sequentially and deliver perforin for cytolytic killing of these targets. Using microscopy to visualize degranulation from individual NK cells, we found that repeated activation via the Fc receptor CD16 decreased the amount of perforin secreted. However, perforin secretion was restored upon subsequent activation via a different activating receptor, NKG2D. Repeated stimulation via NKG2D also decreased perforin secretion, but this was not rescued by stimulation via CD16. These different outcomes of sequential stimulation could be accounted for by shedding of CD16 being triggered by cellular activation. The use of pharmacological inhibitors and NK cells transfected to express a noncleavable form of CD16 revealed that CD16 shedding also increased NK cell motility and facilitated detachment of NK cells from target cells. Disassembly of the immune synapse caused by CD16 shedding aided NK cell survival and boosted serial engagement of target cells. Thus, counterintuitively, shedding of CD16 may positively impact immune responses.

## Abstract (precis)

A long-standing unknown is how an immune synapse disassembles. In this study, Srpan et al. show that shedding of CD16 promotes the detachment of NK cells from target cells to aid serial engagement of multiple targets and to sustain NK cell viability.

## Introduction

Natural Killer (NK) cells are key players of innate immune defense against cancerous or virally infected cells (Vivier et al., 2008, 2011). They can lyse diseased cells directly by secretion of cytolytic granules containing pore-forming perforin and lytic granzymes (Orange, 2008; Voskoboinik et al., 2015) into the synaptic cleft (Cartwright et al., 2014). NK cells also contribute to inflammation more broadly by secreting cytokines including IFN-γ and TNF-α (Fauriat et al., 2010). Their responses are regulated by a variety of germline-encoded activating and inhibitory receptors that serve to elicit a response when appropriate while ensuring tolerance to self.

Activating receptor NK group member D (NKG2D) is one of the best-studied NK cell receptors (Molfetta et al., 2016). It recognizes major histocompatibility complex (MHC) class I chain–related protein A (MICA), MICB, or UL16 binding protein (ULBP) 1–6 proteins that are rarely expressed at the surface of healthy cells but are up-regulated on, for example, tumor-transformed or virally infected cells. NK cells also express the Fc receptor CD16 (FcγRIIIa), which can trigger antibody-dependent cellular cytotoxicity (ADCC) against opsonized cells. ADCC is clinically important as one of the mechanisms of therapeutic antibodies. For anti-CD20 mAb rituximab, widely used for treatment of non-Hodgkin’s lymphoma and autoimmune diseases (Edwards et al., 2004; Cheson and Leonard, 2008), for example, the engagement of Fc receptors has been shown to be vital for its activity in vivo (Clynes et al., 2000).

Tumor infiltrating or blood NK cells isolated from patients with chronic diseases such as HIV commonly display very low levels of activating receptors. This has been associated with decreased NK cell cytotoxicity and increased disease severity (Costello et al., 2002; Groh et al., 2002; Coudert et al., 2005; Wiemann et al., 2005; Konjević et al., 2007). Receptor down-regulation is commonly the result of internalization; NKG2D, for example, undergoes clathrin-mediated endocytosis upon the ligation of membrane-bound or soluble ligands (Ogasawara et al., 2003; Cerboni et al., 2009). Internalized NKG2D along with its signaling adaptor DAP10 can contribute to activating signaling though ERK1/2 (Quatrini et al., 2015). However, internalization also leads to lysosomal degradation of NKG2D, which is thought to be an important physiological response for dampening immune responses that might otherwise be excessive and damaging.

In contrast with NKG2D, down-modulation of CD16 is caused by proteolytic cleavage of its extracellular portion by A disintegrin and metalloproteinase-17 (ADAM17; Romee et al., 2013) or membrane type 6 matrix metalloproteinase (MMP25; Peruzzi et al., 2013). While a proportion of NKG2D can be rapidly recycled back to the cell surface, recovery of CD16 expression is much slower. When CD16 down-regulation was induced by 18 h exposure to seasonal influenza vaccine, its expression only partially recovered by day 18 (Goodier et al., 2016). This suggests that once NK cells are activated, their capacity for ADCC is impaired for several days. The possibility of any beneficial role for shedding of CD16 has not been described other than that it may serve to prevent excessive immune responses.

NK cell activation and the assembly of the immune synapse have been widely studied (Davis et al., 1999; Orange, 2008; Carisey et al., 2018), but how activating signals are terminated and how NK cells dissociate from target cells have been considered far less (Netter et al., 2017). Several lines of research indicate the importance of understanding disassembly of the immune synapse and NK cell detachment. After lysis of one target cell, NK cells can dissociate and move on to discern the state of health of another cell (Martz, 1976; Vanherberghen et al., 2013). Indeed, most target cells die as a result of serial killing (Choi and Mitchison, 2013). In vitro microscopy of NK cells revealed that they can kill up to seven targets in 12 h (Bhat and Watzl, 2007; Vanherberghen et al., 2013). Similarly, in vivo imaging of cytotoxic T lymphocytes (CTLs) has shown that one CTL can kill 2–16 virus-infected cells per day (Halle et al., 2016). Chimeric antigen receptor T cells have recently been reported to exhibit particularly fast off rates from target cells, which may be important in their efficacy (Davenport et al., 2018). In addition, the inhibition of target cell caspases to prevent target cell lysis prolongs contact time with murine NK cells (Jenkins et al., 2015). Such increased engagement leads to further secretion of cytokines and chemokines, which can lead to hyperinflammation. Thus, it is clear that NK cell detachment is important on several levels, yet specific mechanisms for immune cell detachment have not yet been elucidated.

To study sequential activation of NK cells, we designed a microscopy-based assay that allowed us to compare the amount of perforin secreted from individual cells. We found that the order in which cells are activated through different receptors determines the amount of secreted perforin. Specifically, cells sequentially stimulated through CD16 significantly reduced the amount of perforin secreted per cell. Surprisingly, however, subsequent stimulation via NKG2D recovered perforin secretion. When the order of stimulation was reversed, NK cell activation via CD16 could not restore perforin secretion after stimulation through NKG2D. Thus, the specific ligands expressed on target cells are a major factor in the strength of sequential NK cell responses. These results could be accounted for by shedding of CD16, which occurs upon NK cell activation. However, although shedding of CD16 decreased NK cell perforin secretion upon successive contacts, it also proved to be important for the detachment of NK cells from opsonized target cells. Inhibition of shedding CD16 resulted in extended contact times and greater activation-induced death of NK cells. Thus, shedding of CD16 decreases NK cell responses at individual cell–cell contacts but enhances detachment from opsonized target cells, which sustains NK cell survival and increases serial engagement of target cells.

## Results

### Effective restimulation of NK cells is receptor dependent

The predominant mechanism for target cell lysis by NK cells is via the directed secretion of granules containing perforin (Hsu et al., 2016). To compare the amount of perforin secreted per individual cell, we developed a method in which secreted perforin was captured and visualized by confocal microscopy. For this, slides were coated with an anti-perforin mAb as well as other proteins to stimulate NK cells: rituximab, a therapeutic mAb engaging CD16 through its Fc portion, or recombinant human MICA, a ligand for NKG2D. To address the previously described role of ICAM-1 in directed secretion (Hsu et al., 2016), activating ligands were coated in combination with recombinant human ICAM-1 or with Noggin, used as a negative control. Human primary NK cells were incubated on these surfaces for 1 h, after which cells were gently removed, and captured perforin was stained with another, noncompeting Alexa Fluor 488–conjugated anti-perforin mAb for imaging. Both rituximab and MICA triggered secretion of IFN-γ in a dose-dependent manner, confirming their ability to trigger NK cell activation (Fig. S1, A–C).

Visualization of perforin secreted from individual cells revealed that secretion occurred in discrete puncta. Interestingly, perforin secreted from cells stimulated by rituximab commonly appeared smeared, whereas perforin from cells stimulated by MICA formed dense circular clusters (Fig. S1 D). Surfaces coated with no activating ligand induced little degranulation from a few cells, whereas activating surfaces were potent stimulators (Fig. S1, D–F). There was significant variability in the amount of perforin secreted per cell within one donor (Fig. S1 E) as well as between donors (Fig. S1 F). The presence of ICAM-1 did not have a significant impact on degranulation in these conditions. The amount of perforin secreted by each cell increased somewhat over time for cells stimulated by rituximab but less by MICA (Fig. S1, G–J). Concentrations of both ligands were chosen such that secretion of perforin reached a peak (Fig. S2, A–F), and the proportion of cells degranulating after 1 h stimulation was comparable (Fig. S2 G).

**Figure 1.**
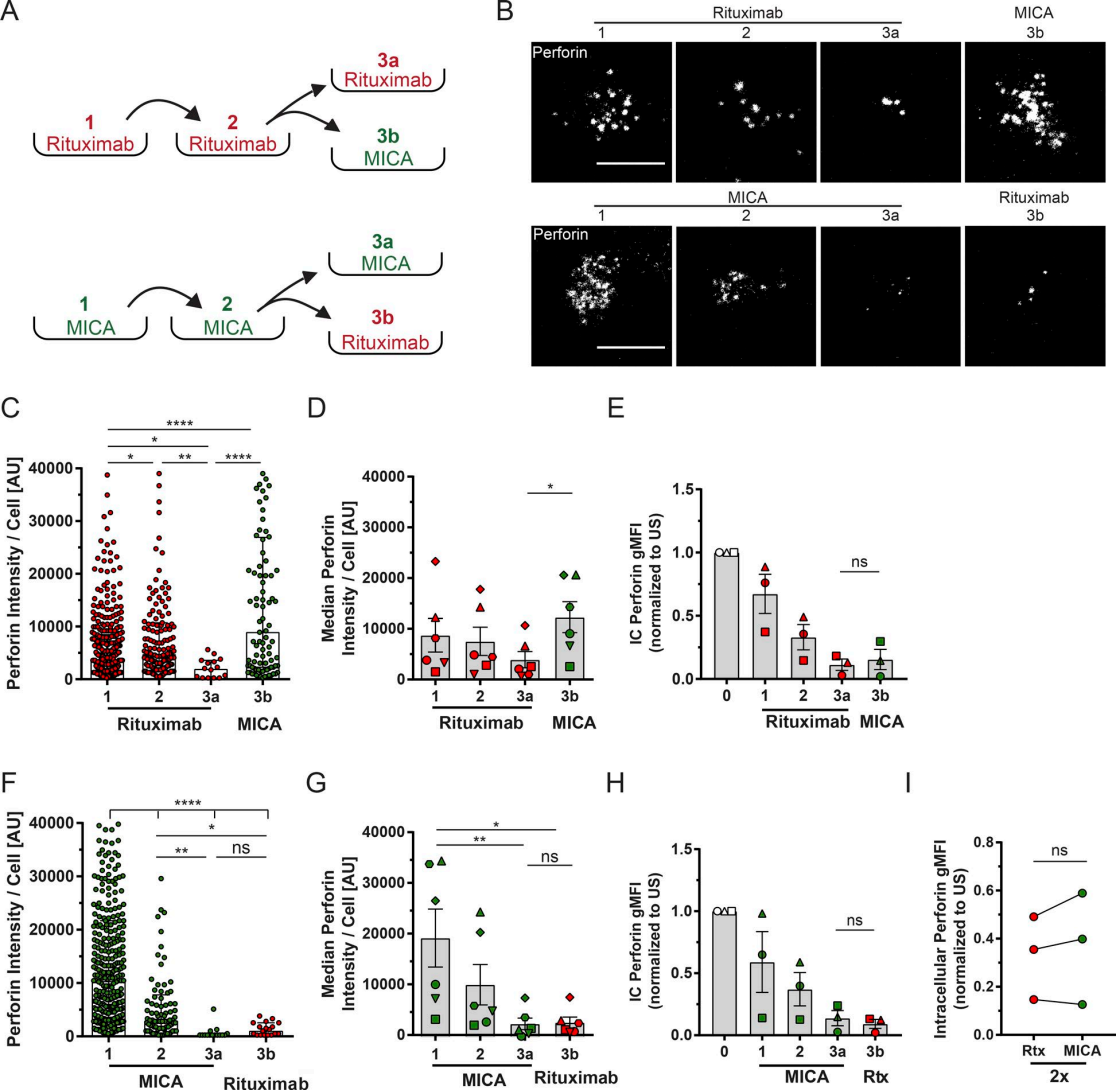
**Effective restimulation of NK cells is receptor dependent. (A–I)** NK cells were sequentially activated through CD16 and NKG2D on slides coated with either rituximab (Rtx) or MICA and both with ICAM-1. Slides were also coated with anti-perforin mAb to capture secreted perforin, which was visualized by a noncompeting Alexa Fluor 488–labeled anti-perforin mAb. **(A)** Schematic representation of experimental approach. NK cells were sequentially incubated for 1 h on differently coated surfaces as indicated. **(B)** Representative images of perforin secreted from one cell during sequential stimulations. Bars, 10 µm. **(C and F)** Quantitative analysis of perforin secreted by cells from a representative donor. Each point is the integrated fluorescence intensity (IFI) of perforin captured from one cell (median ± interquartile range [IQR]). **(D and G)** Median IFI values of perforin secretion from different donors (*n* = 6; mean ± SEM; symbols represent different donors). **(E and H)** Intracellular (IC) perforin levels were assessed by flow cytometry upon each round of stimulation. Graphs represent geometric mean fluorescence intensity (gMFI) values (*n* = 3; mean ± SD; symbols represent different donors). **(I)** Comparison of gMFI values of intracellular perforin upon two rounds of stimulation on either rituximab or MICA. *, P < 0.05; **, P < 0.01; ****, P < 0.0001 calculated by Kruskal-Wallis test (C and F), Friedman test (D, E, G, and H), or Student’s *t* test (I). US, unstimulated. See also Figs. S1, S2, and S3.

In a tumor microenvironment, for example, NK cells encounter target cells expressing a variable repertoire of activating ligands (Ljunggren and Malmberg, 2007; Guillerey et al., 2016), and treatment with a therapeutic mAb will additionally contribute to the diversity of ligands presented by tumor cells. To address how NK cell responses are affected by sequential stimulation, the same cells were incubated on activating surfaces for three sequential 1-h periods. To determine whether the order in which different receptors are triggered impacts sequential degranulation, cells were split into two groups for the final incubation: one group of cells was incubated on the surfaces coated with a repeat of the ligand used for the previous two stimulations, and the other group of cells was introduced to a new ligand (Fig. 1 A). Secreted perforin was captured and imaged after each stimulation. Repeated stimulation on rituximab led to a decrease in perforin secretion per cell. Strikingly, however, upon subsequent stimulation via MICA, secretion of perforin recovered (Fig. 1, B–D). When the order of activation was reversed, secretion of perforin similarly decreased upon each sequential stimulation with MICA, but subsequent activation via rituximab did not recover this (Fig. 1, B, F, and G).

To exclude the impact of the Fc portion of the recombinant MICA protein used in this study, we repeated the experiment using MICA produced with a His tag (MICA-His; Fig. S3, A and B) and compared another ligand for NKG2D, ULBP2 (Fig. S3, C and D). Upon sequential stimulation with MICA-His or ULBP2, perforin secretion decreased and remained low even when cells were subsequently activated via CD16. In addition, MICA-His or ULBP2 could rescue perforin secretion after sequential stimulation via CD16. An anti-CD16 mAb did not rescue degranulation in cells first stimulated twice on rituximab (Fig. S3 E). However, an anti-NKp30 mAb could recover perforin secretion after stimulation with rituximab, indicating that ITAM-mediated signaling machinery used by both CD16 and NKp30 is not impaired or desensitized by serial stimulation (Fig. S3 E).

It has been previously reported that cells undergo exhaustion upon persistent activation (Bi and Tian, 2017). In this study, we found that the amount of intracellular perforin decreased with each stimulation (Fig. 1, E and H). However, as intracellular perforin levels after two stimulations on rituximab or MICA were comparable, this reduction cannot account for the differential outcomes upon a third stimulation with different ligands (Fig. 1 I). Thus, in this specific scenario, cellular exhaustion or depletion of intracellular perforin cannot account for the decrease in perforin secretion seen upon sequential stimulation. Moreover, these data establish that the order in which activating receptors on NK cells are triggered impacts the sequential cytotoxic response.

### Receptor expression varies with serial engagement

**Figure 2.**
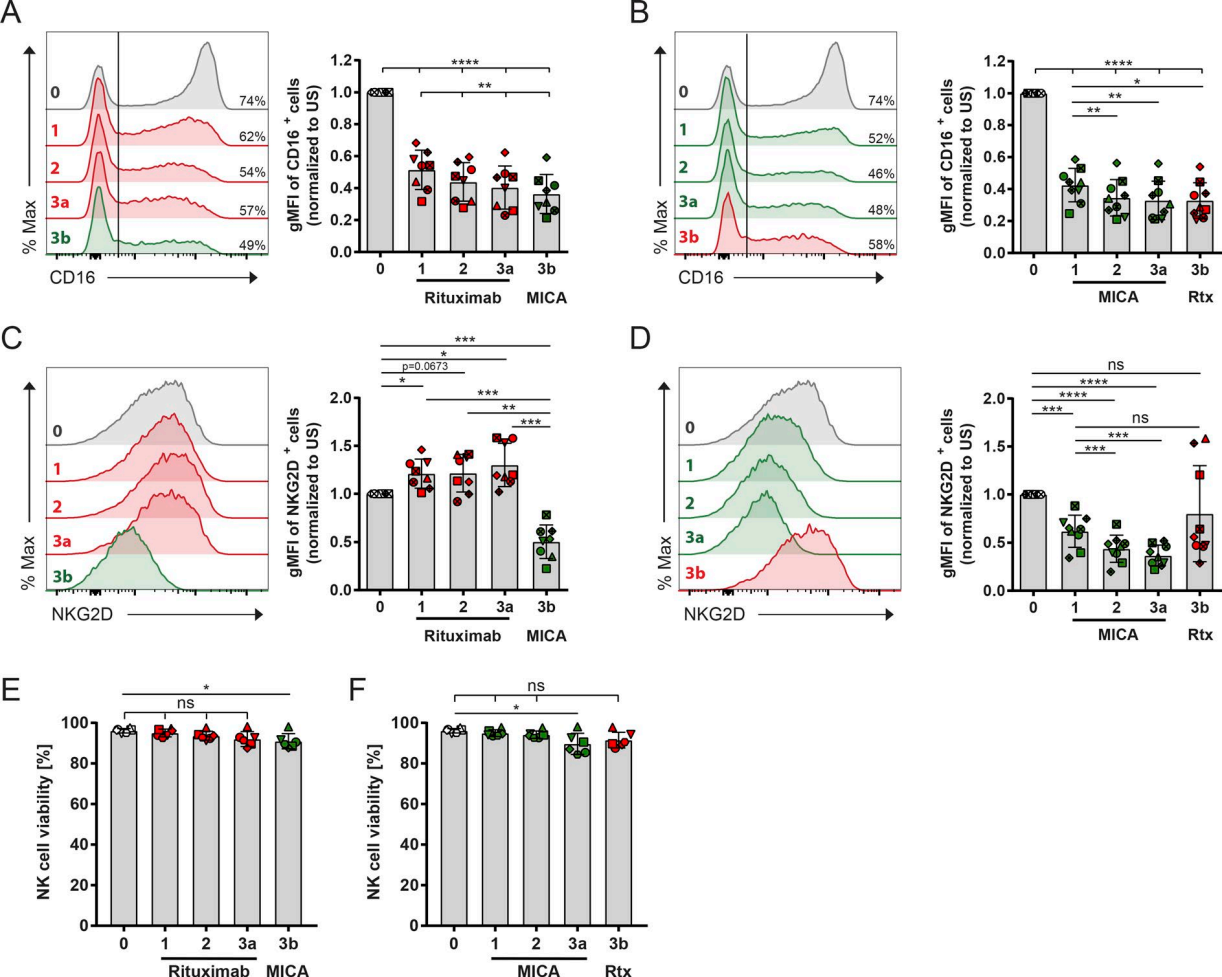
**Receptor expression varies with serial engagement. (A–F)** NK cells were sequentially stimulated through CD16 and NKG2D on surfaces coated with either rituximab (Rtx) or MICA and both with ICAM-1 as schematically represented in Fig. 1 A (1 h at each step). Then, CD16 and NKG2D levels were assessed by flow cytometry. Histograms represent surface expression of CD16 (A and B) and NKG2D (C and D) after each step as indicated. **(A and B)** The vertical line denotes the point at which NK cells were considered as CD16^+^. Numbers denote the percent of CD16^+^ cells from a representative donor. Graphs show the gMFI of CD16 expression of CD16^+^ populations normalized to unstimulated (US) control cells. **(C and D)** gMFI of NKG2D expression from the total NK cell population. **(E and F)** NK cell viability after each stimulation. *n* = 7; symbols represent different donors; mean ± SD. *, P < 0.05; **, P < 0.01; ***, P < 0.001; ****, P < 0.0001 calculated by one-way ANOVA.

We next assessed whether the expression levels of CD16 and NKG2D changed upon stimulation, with a view to testing whether this could account for changes in the NK cell’s functional response. For this, surface receptor expression on cells removed from the coverslips coated with activating NK cell ligands were assessed by flow cytometry. Sequential stimulation with rituximab resulted in a decrease of the surface expression of CD16 (Fig. 2 A). Subsequent stimulation with MICA reduced the expression of CD16 further. Initial activation with MICA also reduced the expression of CD16, and subsequent stimulation through CD16 had little, if any, impact (Fig. 2 B). However, sequential stimulation by rituximab did not negatively affect NKG2D expression; in fact, expression of NKG2D increased slightly (Fig. 2 C). MICA decreased NKG2D expression (Fig. 2 D), and NKG2D levels were restored in some donors when cells were subsequently incubated for 1 h on rituximab, ICAM-1, or in media alone, suggesting that expression of NKG2D recovers within this short time frame independently of stimulation (Fig. S4 A). Consistent with a previous study (Goodier et al., 2016), CD16 levels did not recover for at least 24 h (Fig. S4 B). Confirming that changes in receptor expression were not caused by a particular subset of NK cells being removed from the population, NK cell viability was not affected by cellular activation (Fig. 2, E and F). Thus, receptor expression levels correlated with changes in perforin secretion for subsequent stimulation of NK cells; initial engagement of CD16 did not affect subsequent stimulation via NKG2D, whereas stimulation through CD16 was impaired after activation via NKG2D. This indicates that the order in which different receptors on NK cells are triggered differentially affects NK cell receptor expression, which in turn impacts functional responses.

### CD16 expression changes determine the strength of NK cell serial responses

**Figure 3.**
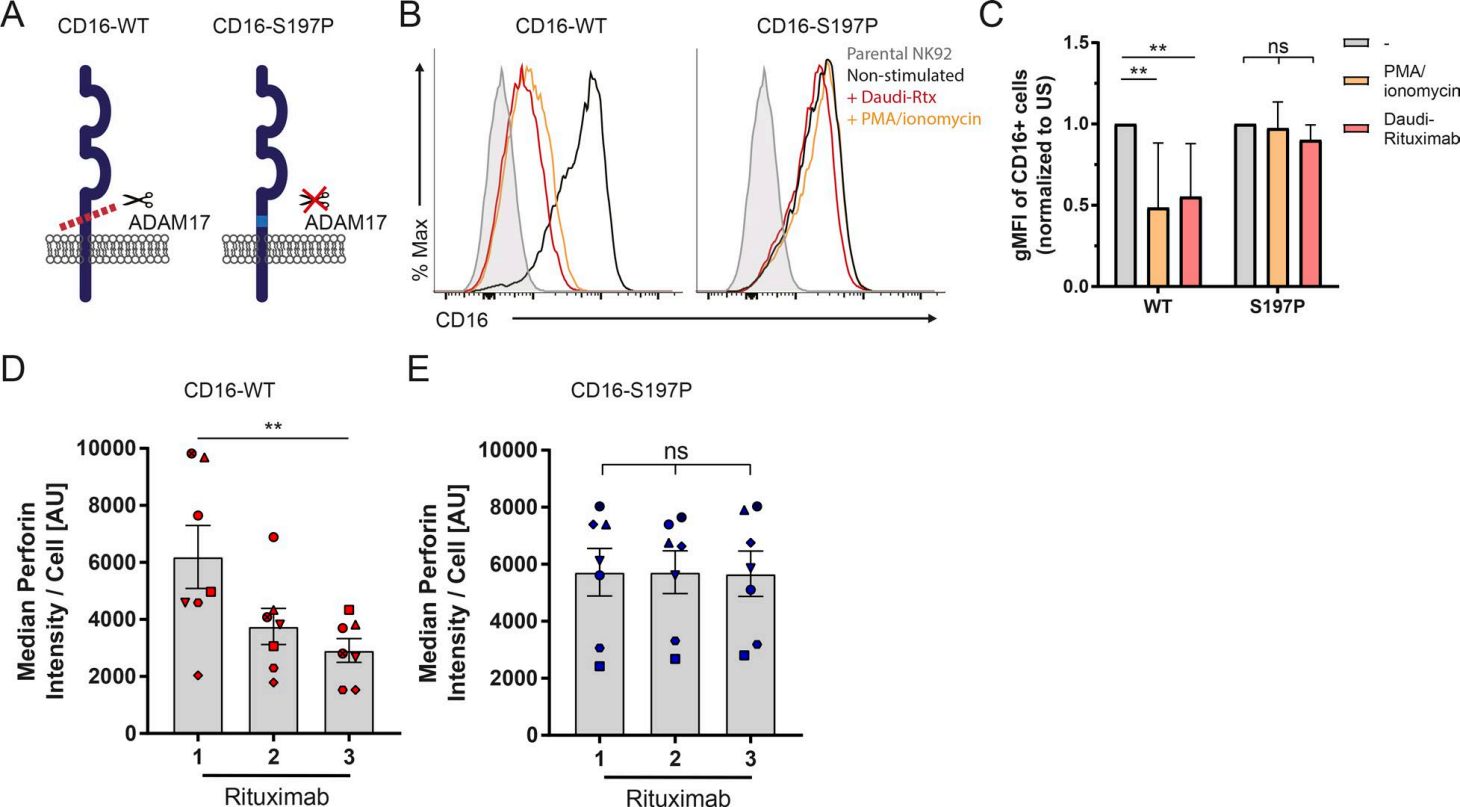
**Expression of a noncleavable form of CD16 prevents reduction of perforin in sequential stimulation. (A)** Schematic representation of CD16-WT and CD16-S197P indicating a single point mutation, S197P, which renders CD16 insensitive to ADAM17. **(B)** The NK92 cell line was transduced to express CD16-WT or CD16-S197P. Representative histograms of surface CD16 on parental cell line (gray) and NK92/CD16-WT or NK92/CD16-S197P before (black) and after activation with Daudi-rituximab (Rtx; red) or PMA/ionomycin (orange). The same histogram for a parental cell line is shown in both panels. **(C)** CD16 expression levels normalized to unstimulated (US) CD16^+^ cells (*n* = 5 independent experiments; mean ± SD). **(D and E)** Transfected NK92 cells were sequentially activated through CD16 on slides coated with rituximab and ICAM-1. Secreted perforin was captured with anti-perforin mAb and visualized by a noncompeting Alexa Fluor 488–labeled anti-perforin mAb. Median fluorescence of perforin for NK92/CD16-WT (D) or NK92/CD16-S197P (E). *n* = 7; mean ± SEM; symbols represent different experiments. **, P < 0.01 calculated by two-way ANOVA (C) or Friedman test (D and E).

To determine whether the reduction of CD16 is responsible for decreased perforin secretion, we transfected NK92, an NK cell line that does not constitutively express CD16, to express WT CD16 (NK92/CD16-WT) or a mutated variant lacking the previously identified cleavage site for ADAM17, in which serine at residue 197 was replaced by proline (NK92/CD16-S197P; Fig. 3 A; Jing et al., 2015). Both variant CD16 proteins were expressed to a similar level at the cell surface (Fig. 3 B). When these transfectants were activated by coincubation with Daudi, a malignant B cell line, opsonized with rituximab (Daudi-rituximab), CD16-WT was efficiently cleaved from the cell surface, whereas levels of CD16-S197P remained intact (Fig. 3 C). For an alternative route of cellular activation independent of ligating CD16 itself, cells were activated by PMA/ionomycin. Again, expression of CD16-WT decreased, whereas CD16-S197P remained intact. When cells were sequentially stimulated on rituximab for 1 h, the amount of perforin secreted from NK92/CD16-WT decreased after each round, whereas degranulation of NK92/CD16-S197P remained unchanged (Fig. 3, D and E). This supports the notion that the receptor expression is a major determinant of NK cell degranulation in sequential stimulations.

### NK cell serial killing of target cells expressing different ligands is determined by the order of engagement

We next set out to assess whether decreased perforin secretion upon sequential stimulations impacts the killing of target cells. For this, interactions with Daudi, Daudi-rituximab, or Daudi transfected to express MICA at the cell surface (Daudi-MICA) were compared (Fig. S4, C and D). 1 h incubation with Daudi, Daudi-rituximab, or Daudi-MICA did not significantly affect NK cell viability (Fig. S4 E), but it lowered NK cell expression of CD16 (Fig. S4, F–H). NKG2D levels, however, were only affected when this receptor was engaged by Daudi-MICA and less significantly by Daudi alone (which constitutively expresses a low level of NKG2D ligands; Fig. S4, I and J). Daudi-rituximab did not have any additional effect on NKG2D expression. To test whether these results were specific to Daudi, we also used 721.221 (hereafter referred to as 221), an EBV-transformed B cell line selected to lack expression of class I MHC proteins. 221 is susceptible to NK cell–mediated lysis on account of it lacking class I MHC protein, and thus it is killed according to the missing-self hypothesis. NK cells were incubated with 221 or 221 transfected to express MICA (221-MICA), or they were opsonized with rituximab (221-rituximab), and in either case, the presence of these target cells efficiently caused NK cell loss of CD16 (Fig. S4 K). However, when NK cells were incubated with P815, a cell line that does not express any ligands for NK cell receptors, the levels of surface CD16 were not significantly affected. Thus, the loss of CD16 from NK cell surfaces is not something triggered only by Daudi cells, and moreover, other triggers of NK cell lysis, e.g., missing self, lead to loss of CD16. Thus, these data with target cells are consistent with results obtained from activating surfaces and further indicate that decreased expression of CD16 happens upon NK cell activation, whereas decreased expression of NKG2D only occurs with ligation of NKG2D.

**Figure 4.**
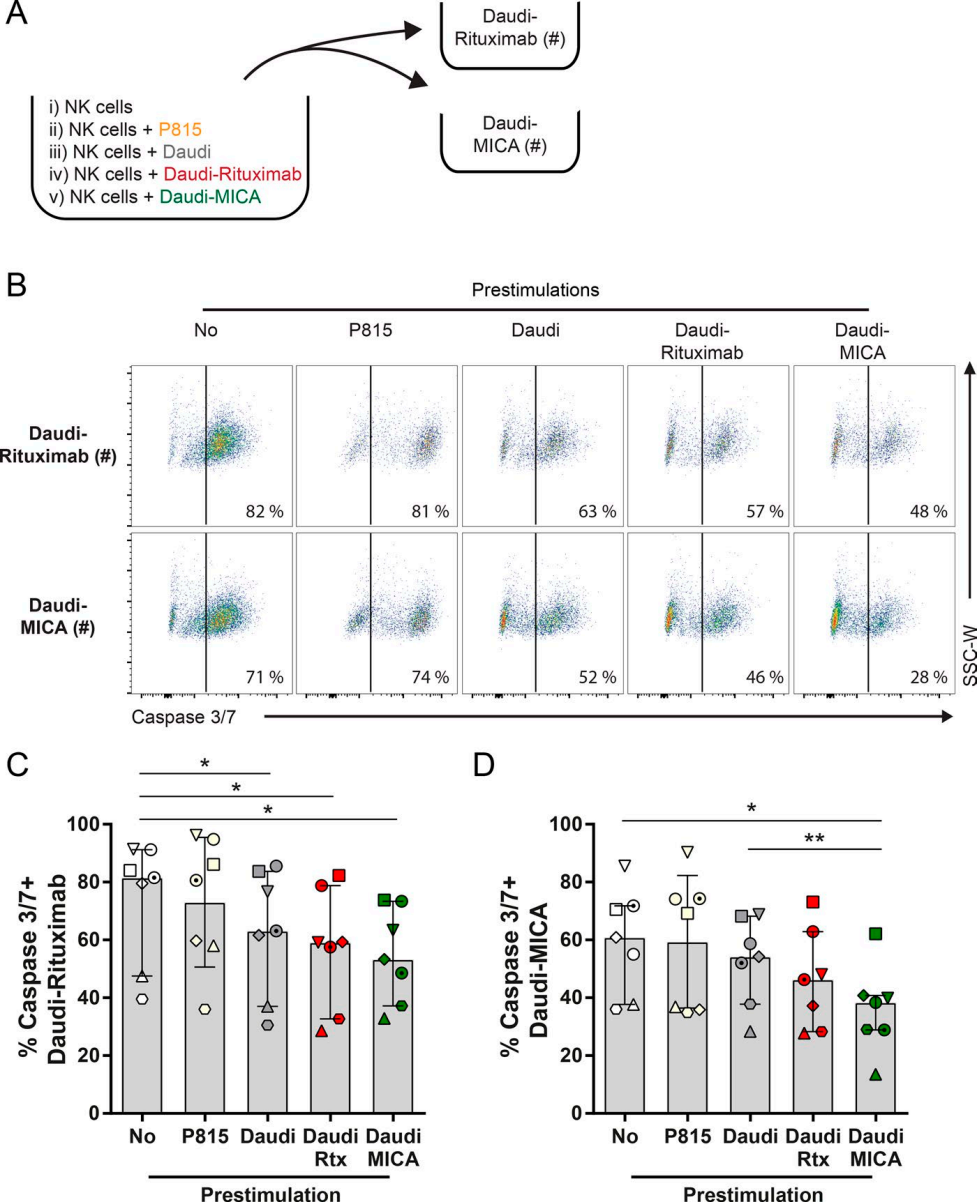
**NK cell prestimulation can affect subsequent target cell lysis. (A–D)** Primary NK cells were prestimulated with P815, Daudi, Daudi-rituximab (Rtx), or Daudi-MICA. Unstimulated NK cells were used as a control. After 1.5 h incubation, Daudi-rituximab or Daudi-MICA cells labeled with cell trace dye (#) were added and incubated for an additional 1.5 h. **(A)** Schematic representation of experimental setup. **(B)** Representative plots of target cell death measured by staining caspase 3/7 activity and assessed by flow cytometry gated on labeled target cells (#). Percentage of caspase 3/7–positive Daudi-rituximab (C) and Daudi-MICA (D). *n* = 7; mean ± SD; symbols represent different donors. *, P < 0.05; **, P < 0.01 calculated by one-way ANOVA. SSC-W, side scatter–width. See also Fig. S4.

It has recently been established that as little as two to four degranulation events suffice to trigger target cell lysis (Gwalani and Orange, 2018). Thus, to address whether receptor down-regulation and reduced perforin secretion affect NK cell cytotoxicity in serial killing, we first preactivated NK cells with P815 or Daudi, Daudi-rituximab, or Daudi-MICA. After 1.5 h, additional target cells labeled with a dye were added for an additional 1.5 h in the presence of a second dye, indicating cell death by staining caspase 3/7 (Fig. 4 A). Unstimulated NK cells killed 74 ± 21% of Daudi-rituximab and 60 ± 18% of Daudi-MICA. Preactivation with P815 did not affect subsequent killing of Daudi-rituximab (73 ± 22%) or Daudi-MICA (59 ± 23%), whereas the preactivation with either target cell (Daudi, Daudi-rituximab, or Daudi-MICA) significantly reduced the subsequent killing of Daudi-rituximab (by 15%, 23%, or 26% respectively; Fig. 4, B and C). Killing of Daudi-MICA was only significantly decreased when NK cells were preactivated with Daudi-MICA (by 40%; Fig. 4, B and D). These data indicate that the order of serial engagement of target cells impacts the outcome of NK cell surveillance. However, in a bulk assay like this, it is not clear how many target cells each individual NK cell has contacted.

**Figure 5.**
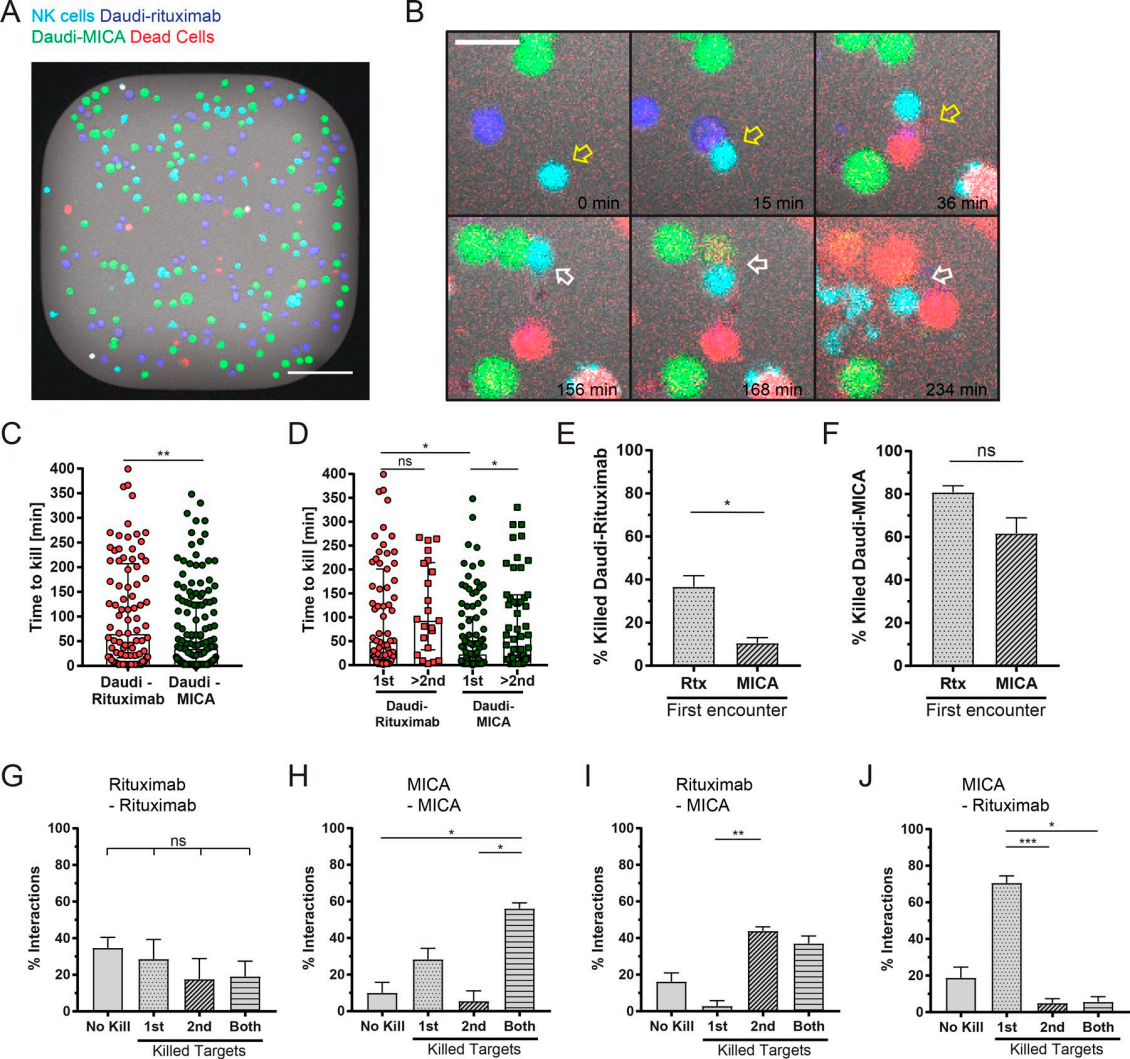
**NK cell serial killing of target cells expressing different ligands is determined by the order of engagement. (A–J)** NK cells were incubated with both Daudi-rituximab and Daudi-MICA at an E:T:T of 1:1.5:1.5 in microwells. Images were captured every 3 min for 8 h. **(A)** Representative image of one well (450 × 450 µm^2^) in fluorescence overlaid onto brightfield. NK cells are shown in cyan, Daudi-rituximab (Rtx) in blue, Daudi-MICA in green, and dead cells in red. Bar, 100 µm. **(B)** Representative images of enlarged regions from one microwell with time indicated. Bar, 20 µm. The example sequence shows how an NK cell (cyan) contacted a Daudi-rituximab (blue, 15 min) and killed it (36 min; yellow arrow). The NK cell detached, encountered a Daudi-MICA (green, 156 min) and killed it (168 min; target cell loses green fluorescence and begins to turn red; white arrow). Both targets were dead (red), whereas the NK cell remained alive (234 min). **(C)** Time required to lyse Daudi-rituximab and Daudi-MICA from initial contact (median ± IQR). **(D)** Time for target cell lysis stratifying initial and successive kills (median ± IQR). **(E)** Percentage of target cell lysis when Daudi-rituximab was encountered after contact with another Daudi-rituximab or Daudi-MICA. **(F)** Percentage of Daudi-MICA lysis when Daudi-MICA was met after Daudi-rituximab or Daudi-MICA. **(G–J)** Time-lapse microscopy was analyzed to record the outcome of different combinations of sequential interactions. Either no targets were killed (No kill), only the first (1st) or only the second (2nd) target was killed, or both were killed. Interactions that were unclear, e.g., many cells clustered together, were excluded from analysis. Graphs show the percentage of target cells killed from the following sequential encounters; (G) Daudi-rituximab then Daudi-rituximab, (H) Daudi-MICA then Daudi-MICA, (I) Daudi-rituximab then Daudi-MICA, and (J) Daudi-MICA then Daudi-rituximab. *n* = 231 interactions from three independent experiments; mean ± SD. *, P < 0.05; **, P < 0.01; ***, P < 0.001 by Mann Whitney test (C), Kruskal-Wallis test (D), Student's *t* test (E and F), or one-way ANOVA (G–J).

To more accurately scrutinize serial cytotoxicity of individual NK cells, we used live-cell time-lapse microscopy within specially designed microchips (Guldevall et al., 2010; Varadarajan et al., 2011; Vanherberghen et al., 2013), allowing the tracking of individual NK cell–target cell interactions over several hours (Fig. 5, A and B). NK cells and target cells were each labeled with different dyes, and cell death was visualized by the loss of the initial fluorescence as well as by an accumulation of a dead cell stain. Overall, killing of Daudi-rituximab was slower than killing of Daudi-MICA (median time after first contact was 66 min and 36 min, respectively; Fig. 5 C). However, the first kill was executed faster than sequential kills (Fig. 5 D; statistically significant only for Daudi-MICA). When Daudi-rituximab was the first target cell killed, the median killing time was 51 min, whereas for Daudi-MICA, it was 24 min. Killing of Daudi-rituximab subsequently took a median time of 95 min, whereas lysis of Daudi-MICA subsequently took 63 min.

When an NK cell encountered two Daudi-rituximab cells sequentially, the second Daudi-rituximab was killed 37 ± 9% of the time (Fig. 5 E). When Daudi-MICA was the first target cell encountered, subsequent killing of Daudi-rituximab was reduced to only 11 ± 4%. However, when the NK cell had killed a Daudi-rituximab first, killing of subsequent Daudi-MICA occurred in 81 ± 5% (Fig. 5 F). This was not significantly reduced to 62 ± 13% when the preceding contact was with Daudi-MICA.

We next analyzed the sequence of NK cell interactions to compare the outcome of all possible combinations. When an NK cell encountered two Daudi-rituximab cells sequentially, the likelihood of either one or both target cells being killed was similar (Fig. 5 G). When an NK cell encountered multiple Daudi-MICA cells, both cells were most often killed (Fig. 5 H). Contacting Daudi-rituximab then Daudi-MICA, either both target cells were killed (37 ± 7%) or only the second (44 ± 4%; Fig. 5 I). In contrast, when an NK cell first encountered Daudi-MICA and then Daudi-rituximab, the killing of the second target cell was impaired. Only the first target was killed in 71 ± 7% of interactions, whereas both targets were killed 6 ± 5% of the time (Fig. 5 J). Thus, NK cells that ligate CD16 first display relatively unchanged responses toward a second target cell expressing MICA, whereas NK cells that engage NKG2D then present decreased cytotoxicity toward rituximab-opsonized target cells.

### Activation via CD16 triggers the assembly of a cytolytic kinapse

**Figure 6.**
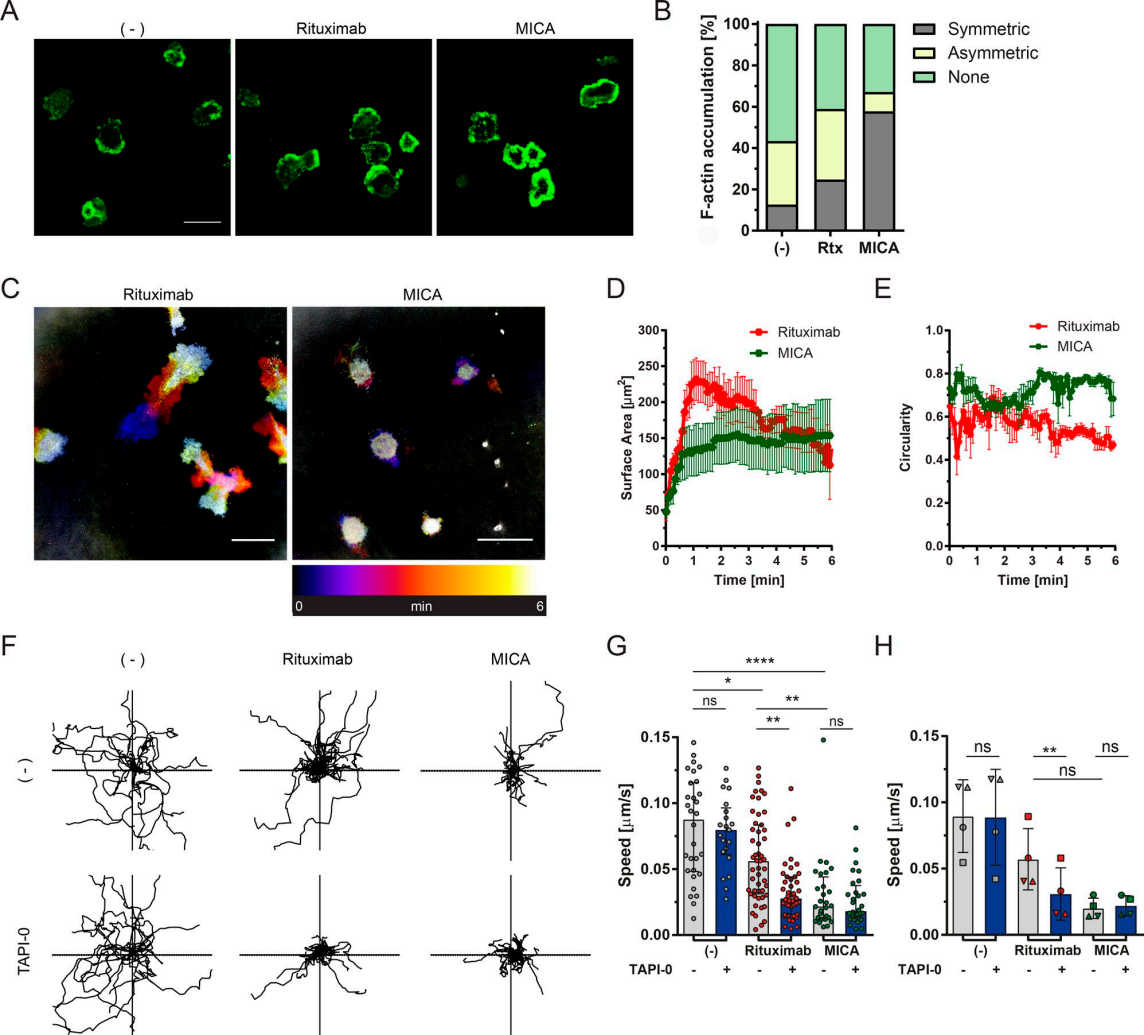
**Activation via CD16 triggers the assembly of a cytolytic kinapse. (A)** NK cells were incubated for 5 min on slides coated with rituximab (Rtx) or MICA, both with ICAM-1, or ICAM-1 alone (−) and then fixed. Panels show representative confocal images of F-actin stained with Alexa Fluor 488–labeled phalloidin. **(B)** Cells were scored according to their F-actin distribution; dense symmetrical rings (gray) accumulated asymmetrically at the leading edge (yellow) or more evenly distributed across the interface (green). *n* = 3 independent experiments. **(C)** IRM live-cell imaging of the contact between NK cells and glass slides coated with rituximab or MICA, both with ICAM-1. An overlay of all 360 frames (6 min at one frame per second) is shown colored according to time. Bars, 20 µm. **(D and E)** Surface area (D) and circularity (E) of cells was analyzed. Circularity values approaching 1 indicate a more circular shape, whereas 0 indicates an elongated shape (*n* = 3 independent experiments; mean ± SEM). **(F)** NK cell motility on activating surfaces. NK cells were labeled with Calcein and allowed to settle on surfaces coated with ICAM-1 alone (−), rituximab, or MICA, both with ICAM-1 ±1 µM TAPI-0 (where indicated). Images were acquired every 30 s for 45 min, and cells were tracked using Cell Tracker, a MATLAB plugin. *n* = 20 individual cell tracks from one representative donor. Axes are ±300 µm. **(G)** Speed of NK cells on differently coated surfaces. Each point represents the average speed of an individual cell from a representative donor (median ± IQR). **(H)** Mean NK cell speed (*n* = 4 different donors; mean ± SEM). *, P < 0.05; **, P < 0.01; ***, P < 0.001; ****, P < 0.0001 calculated by Kruskal-Wallis test (G) or one-way ANOVA (H). See also Fig. S5 and Videos 1 and 2.

To investigate the impact of shedding CD16 on NK cell morphology, cells were incubated on activating surfaces for 5 min to allow cell spreading and synapse formation before they were fixed and stained for F-actin. Confocal microscopy revealed that activation of cells through MICA-coated surfaces triggered accumulation of F-actin into a dense peripheral ring (Fig. 6, A and B) as reported previously (Culley et al., 2009). Only a small proportion of NK cells on control surfaces coated with ICAM-1 alone exhibited a dense ring of F-actin. Surprisingly, activation through rituximab also did not lead to a symmetrical ringed accumulation of F-actin. Similar to ICAM-1, cells on rituximab displayed a more migratory phenotype with an accumulation of F-actin at the leading edge of the cell. Cells on surfaces coated with anti-CD16 mAb also displayed an asymmetrical F-actin distribution (Fig. S5 A). However, other NKG2D ligands such as MICA-His, ULBP2, or anti-NKG2D mAb, all structurally different, or an anti-NKp30 mAb engaging an alternative activating receptor all triggered the formation of stable symmetrical synapses. Allowing cells on rituximab to spread for longer did not change their morphology (Fig. S5 B). This establishes that the type of activating receptor being ligated is a major factor in determining the stability of the NK cell synapse.

To further address the formation of the immune synapse on coated surfaces, we performed time-lapse internal reflection microscopy (IRM). IRM is a label-free technique that can determine the lifetime of dynamic contacts made by cells interacting with coated glass coverslips. On rituximab, NK cells evidently spread more than on surfaces coated with MICA (Fig. 6, C and D). On both surfaces, the maximal area of the interface occurred at ∼1 min. Cells stimulated on MICA maintained a similar size of symmetrical contact over time, whereas cells on rituximab spread with a migratory elongated shape (Fig. 6 E). Cells on rituximab often had long thin tails, the area of which were excluded from analysis and likely contributed to an apparent decline in the cell’s contact footprint seen after 2 min. Importantly, cells interacting with MICA were static and did not move far from the original point of landing, whereas cells on rituximab were much more motile (Fig. 6 C and Videos 1 and 2). This indicates that the synapse formed via NKG2D is considerably more stable than that triggered by CD16.

Track plots also revealed that cells moved significantly on ICAM-1– and rituximab-coated surfaces but were relatively static on MICA (Fig. 6 F). On surfaces coated with rituximab and MICA, at concentrations that lead to similar proportions of cells degranulating (Fig. S2 G), cells moved faster on rituximab (0.058 ± 0.033 µm/s) compared with cells stimulated by MICA (0.030 ± 0.028 µm/s; Fig. 6 G). If many donors were analyzed together, there was considerable variability in the speed of cells moving such that statistical difference between cells moving on rituximab (0.065 ± 0.021 µm/s) versus MICA (0.023 ± 0.007 µm/s) was not reached (Fig. 6 H). There was no evidence that concentration of rituximab could impact the stability of the synapse, whereas the cell arrest on MICA scaled with concentration of ligand (Fig. S5, C–F).

To establish the role of CD16 shedding on NK cell motility on these surfaces, an inhibitor of ADAM17, TAPI-0 was added to cells at a concentration that inhibited shedding of CD16 but had no effect on cell viability or NKG2D expression (Fig. S5, G–M). Inhibition of CD16 shedding did not affect CD16 expression of unstimulated cells nor NK cell degranulation, but it increased IFN-γ production (Fig. S5, K, N, and O) in agreement with previous work (Romee et al., 2013). The addition of TAPI-0 led to significant decrease in cell motility on rituximab but did not affect their motility on ICAM-1 or MICA (Fig. 6, F–H). The average cell speed on rituximab of cells from multiple donors was reduced by TAPI-0 by 44% to 0.036 ± 0.017 µm/s. These data are consistent with ADAM17-induced cleavage of CD16 facilitating the motility of NK cells. Since cells on rituximab did not form stable synapse but did degranulate, we propose that the contact interface formed between NK cells and surfaces coated with rituximab is a cytolytic kinapse.

### Cleavage of CD16 promotes NK cell detachment from rituximab-opsonized target cells

**Figure 7.**
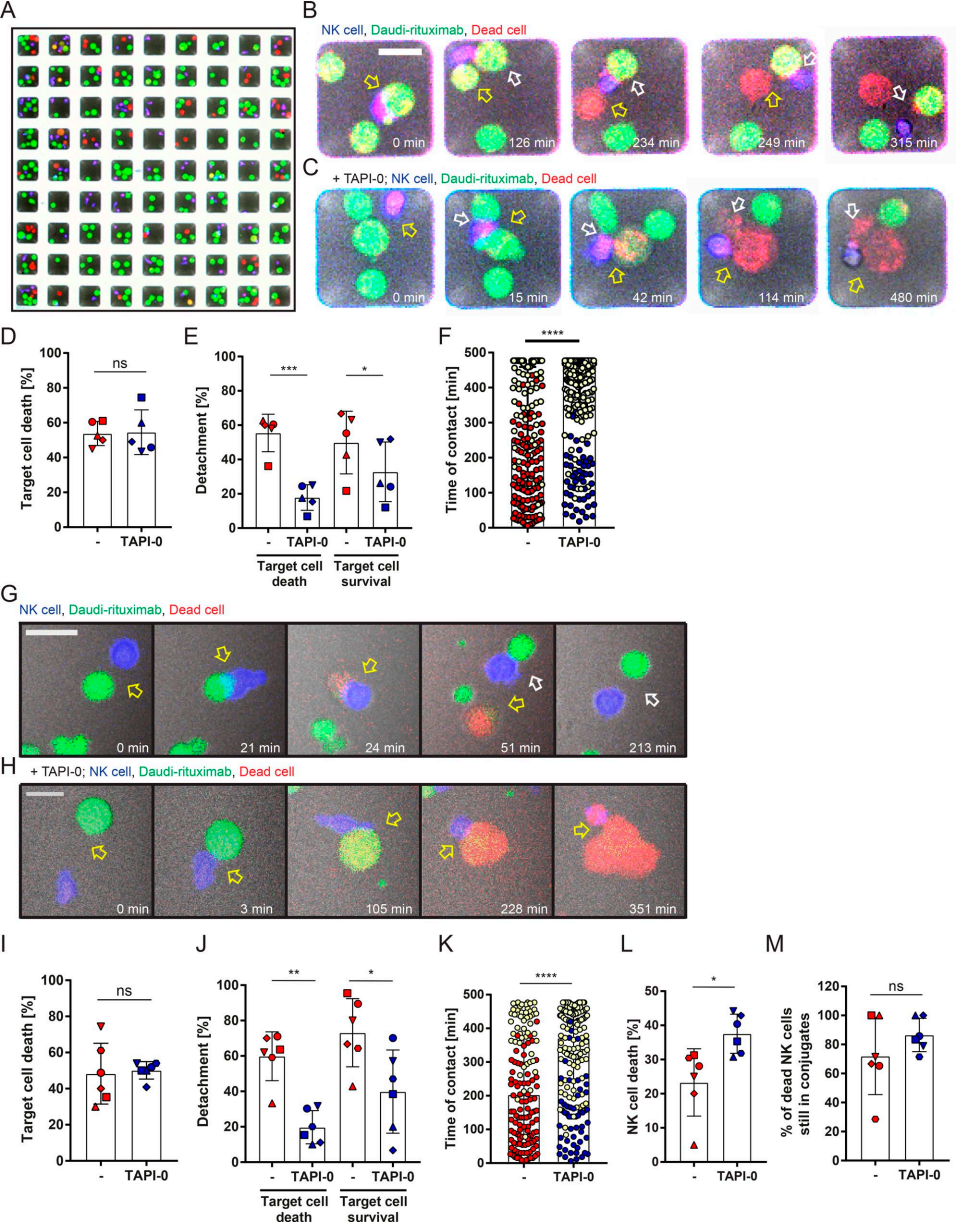
**Inhibition of CD16 shedding impairs detachment of NK cells from opsonized target cells. (A–M)** NK cells were incubated in microwells (50 × 50 µm^2^ [A–F] or 450 × 450 µm^2^ [G–M]) with Daudi-rituximab ± TAPI-0 and imaged every 3 min for 8 h. Images show fluorescence and brightfield images overlaid. **(A)** Representative image of 81 wells (50 × 50 µm^2^) showing NK cells (blue), Daudi-rituximab (green), and dead cells (red). Yellow indicates cells beginning to die. **(B and C)** Representative time-lapse images of individual wells at times indicated. Bars, 20 µm. **(B)** NK cell (blue) formed a conjugate with a target cell (green; 0 min; yellow arrow). The NK cell formed a contact with another target (white arrow, 126 min). First target cell was killed (234 min), the NK cell detached from killed cell (249 min) and detached from the second target without killing (315 min). **(C)** In the presence of TAPI-0, an NK cell contacted two targets (15 min; yellow and white arrows), and both were killed (42 and 114 min), but the NK cell was unable to detach (480 min). **(D)** Percentage of interactions that resulted in target cell lysis (mean ± SD). **(E)** Percentage of NK cells that detached from target cells whether or not the target cell was lysed (mean ± SD). **(F)** The duration of all cytolytic NK cell–target cell contacts. Each point represents an individual contact. Contacts still intact at the end of the acquisition (480 min) are plotted in yellow. These points underestimate the true contact time (median ± IQR). *n* = 886 interactions from five independent experiments; symbols indicate different donors. **(G and H)** Representative time-lapse zoomed-in regions from 450 × 450 µm^2^ microwells. Bars, 20 µm. **(G)** An NK cell (blue) formed a contact with Daudi-rituximab (green; 21 min; yellow arrow). After target cell lysis (24 min), the NK cell detached and moved onto a second target (51 min; white arrow). The second target was not killed, and the NK cell detached (213 min). **(H)** With TAPI-0, an NK cell contacted a target cell (3 min; yellow arrow). After target cell lysis (105 min), the NK cell did not detach and itself died (351 min). **(I)** Percentage of all NK cell–target cell interactions that resulted in target cell lysis. **(J)** Percentage of NK cells that detached from Daudi-rituximab cells whether or not the target cell was lysed. **(K)** Average time that NK cells were in contact with a target cell in which the target cell was lysed. Conjugates that remained attached at the end of the acquisition are plotted in yellow (median ± IQR; each point represents an individual contact). **(L)** Percentage of NK cells that died after target cell contact. **(M)** Percentage of NK cells that died after failing to detach. Mean ± SD; symbols indicate different donors. *n* = 705 interactions from six independent experiments. *, P < 0.05; **, P < 0.01; ****, P < 0.0001 calculated by Student’s *t* test (D, I, L, and M), one-way ANOVA (E and J), or Mann-Whitney test (F and K). See also Videos 3 and 4.

Given that cleavage of CD16 affected NK cell motility on slides, we next set out to test whether shedding of CD16 may also impact NK cell detachment from target cells. For this, we performed live imaging of NK cells interacting with Daudi-rituximab for 8 h in microchips containing multiple wells of dimensions 50 × 50 µm^2^ with one field of view containing 81 wells (Fig. 7, A–C; and Videos 3 and 4). NK cells killed 54 ± 7% of Daudi-rituximab (Fig. 7 D). The addition of TAPI-0 did not affect this outcome (55 ± 13%). Strikingly, in the presence of TAPI-0, detachment was reduced by 67% so that only 18 ± 7% NK cells were able to leave the lysed target cell (Fig. 7 E). Those that did detach took far longer to do so (Fig. 7 F). With TAPI-0 treatment, the majority of NK cells and target cells were still in contact after 8 h (the duration of acquisition; yellow data points in Fig. 7 F).

However, in these small confined wells, the movement of NK cells and target cells was very limited. To avoid the possibility that such restricted movements may impact intercellular contact times, we next used larger wells (450 × 450 µm^2^), allowing more interactions to occur (Fig. 7, G and H). Similar to small wells, 48 ± 17% of interactions led to target cell death in larger wells, and this was not affected by the presence of TAPI-0 (Fig. 7 I). Most importantly, detachment from target cells was again significantly impaired when TAPI-0 was added (Fig. 7 J). Without TAPI-0, 60 ± 14% NK cells detached from lysed targets, and 73 ± 19% detached from target cells that were not killed. When TAPI-0 was added, only 20 ± 9% of NK cells were able to detach from killed Daudi-rituximab, and 40 ± 24% detached from targets surviving the contact. For NK cells that did detach, the inhibitor of CD16 shedding prolonged the contact time (Fig. 7 K). Overall, these data establish that an inhibitor of CD16 shedding, TAPI-0, prevented NK cells being able to detach from target cells.

TAPI-0 does not affect NK cell viability directly (Fig. S5 M). Live-cell imaging, however, revealed that the addition of TAPI-0 leads to significant increase in NK cell death caused by intercellular contact with target cells (Fig. 7 L). Although 23 ± 10% of NK cells in contact with a target cell died over the 8-h acquisition, the presence of TAPI-0 increased this to 38 ± 6%. The majority of NK cell death occurred when NK cells failed to detach from their target (Fig. 7 M). Shedding of surface receptors is therefore not only an important mechanism for the detachment from target cells but also for NK cell survival.

**Figure 8.**
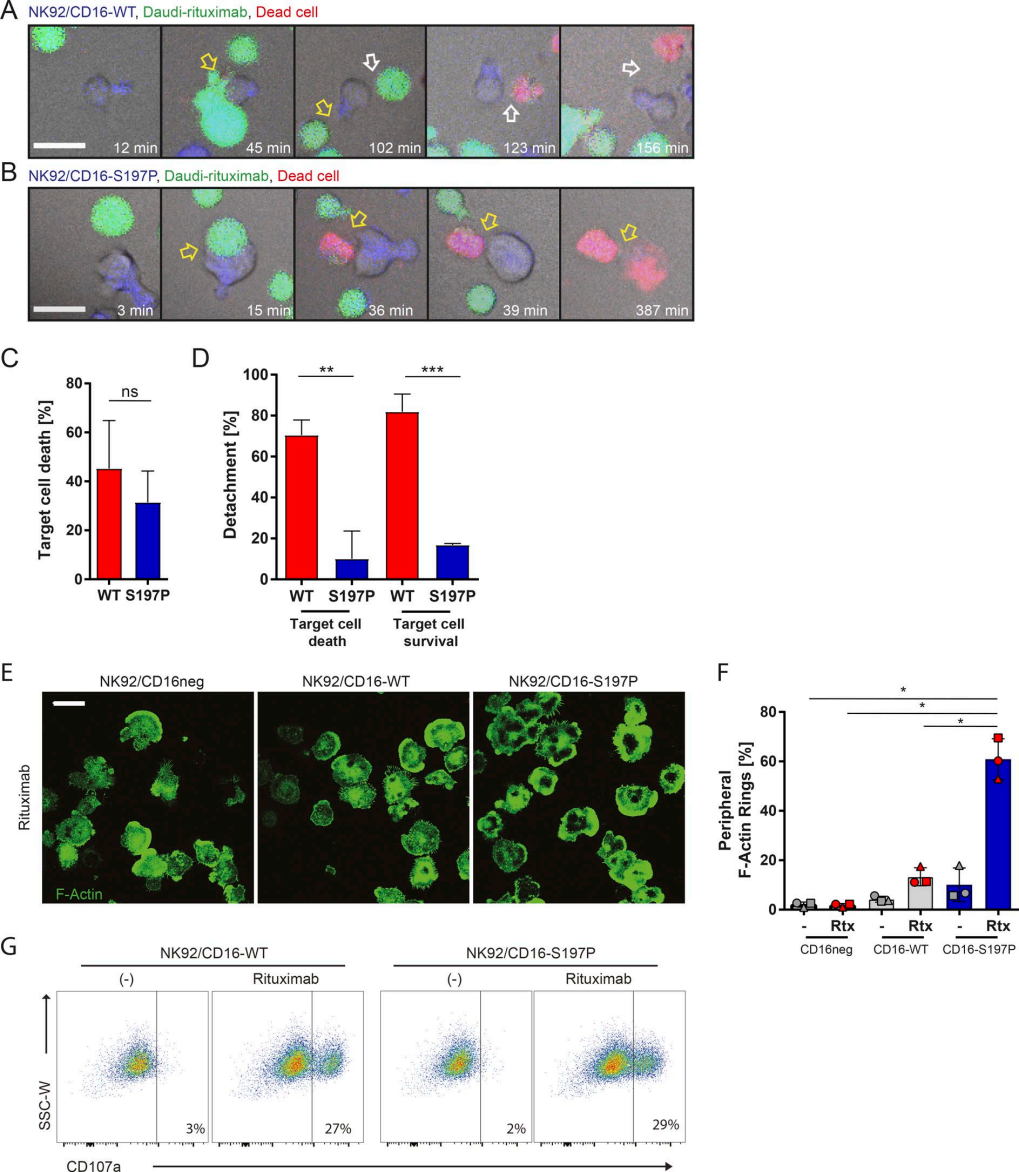
**Expression of a noncleavable form of CD16 leads to prolonged contacts with opsonized target cells. (A and B)** NK92/CD16-WT or NK92/CD16-S197P cells were incubated with Daudi-rituximab (Rtx) in 450 × 450 µm^2^ microwells. Representative time lapse–enlarged regions show composite fluorescence and brightfield images. NK cells (blue), Daudi-rituximab (green), and dead cells (red) at times indicated. Bars, 20 µm. **(A)** An NK92/CD16-WT cell (blue) formed a contact with Daudi-rituximab (green; 45 min; yellow arrow). The NK92 cell detached without killing and established a contact with a new target (102 min; white arrow). The second target cell was killed (123 min), and the NK92 cell was detached (156 min). **(B)** An NK92/CD16-S197P cell formed a contact with a target cell (15 min; yellow arrow) and killed it (36 min). The NK92/CD16-S197P cell died while still attached (387 min). **(C)** Percentage of NK cell–target interactions that resulted in target cell lysis. **(D)** Percentage of NK cells that detached by the end of the acquisition. *n* = 195 interactions from three independent experiments (mean ± SD). **(E and F)** NK92 cells and transfectants were incubated on surfaces coated with ICAM-1 (−) or ICAM-1 with rituximab for 5 min. Cells were fixed and stained with Alexa Fluor 488–labeled phalloidin marking F-actin. **(E)** Representative images of spreading on rituximab with ICAM-1. Bars, 20 µm. **(F)** Percentage of NK92 cells forming dense peripheral rings of F-actin. *n* = 3 independent experiments (mean ± SD). **(G)** Representative dot plot assessing the degranulation marker, CD107a, by flow cytometry. NK92 cells and transfectants were activated on ICAM-1 (−) or rituximab with ICAM-1 for 4 h. **, P < 0.01; ***, P < 0.001 calculated by Student’s *t* test (C) or one-way ANOVA (D and F). SSC-W, side-scatter–width.

TAPI-0 can also inhibit shedding of other proteins from NK cells. Thus, to address whether the shedding of CD16 is the main factor behind these functional responses, we used NK92 cells transfected to express CD16-WT or uncleavable CD16-S197P. Single-cell interactions between opsonized targets (Daudi-rituximab) and NK92 transfectants were imaged in microwells for 8 h (Fig. 8, A and B). The killing efficiency was slightly but not significantly reduced for NK92/CD16-S197P (NK92/CD16-WT killed 45 ± 20% of the encountered targets, and NK92/CD16-S197P killed 31 ± 13%; Fig. 8 C). Most importantly, regardless of whether the target cell survived or was killed, detachment of NK92/CD16-S197P from target cells was significantly impaired (Fig. 8 D). Only 10 ± 14% NK92/CD16-S197P cells detached from killed target cells, compared with 70 ± 7% for NK92/CD16-WT. Similarly, 16 ± 1% of NK92/CD16-S197P detached from target cells that survived the contact, compared with 81 ± 9% for NK92/CD16-WT. These results together establish that CD16 shedding is needed for efficient detachment from opsonized target cells.

We next set to assess how this mutation in CD16 affects synapse formation. NK92 cells and transfectants were allowed to spread on slides coated with ICAM-1 or ICAM-1 plus rituximab for 5 min before being fixed and stained with phalloidin to visualize the organization of F-actin. Very few cells formed dense symmetrical rings of F-actin on surfaces coated with ICAM-1 only. A small fraction of NK92 and NK92/CD16-WT formed rings of F-actin on rituximab (2 ± 0.1% and 11 ± 0.2%, respectively; Fig. 8, E and F). Strikingly, however, far more NK92/CD16-S197P formed dense F-actin rings (65 ± 7%). This provides further evidence that cleavage of CD16 is important for NK cell motility and the assembly of a kinapse, whereas a lack of CD16 cleavage leads to the formation of a stable symmetrical synapse. Because both transfectants degranulated to a similar extent (Fig. 8 G), F-actin rings and stable synapses are not prerequisite for granule release triggered by rituximab. Together, these data establish that a major beneficial impact of CD16 shedding on NK cell responses is its contribution to NK cell detachment and survival.

**Figure 9.**
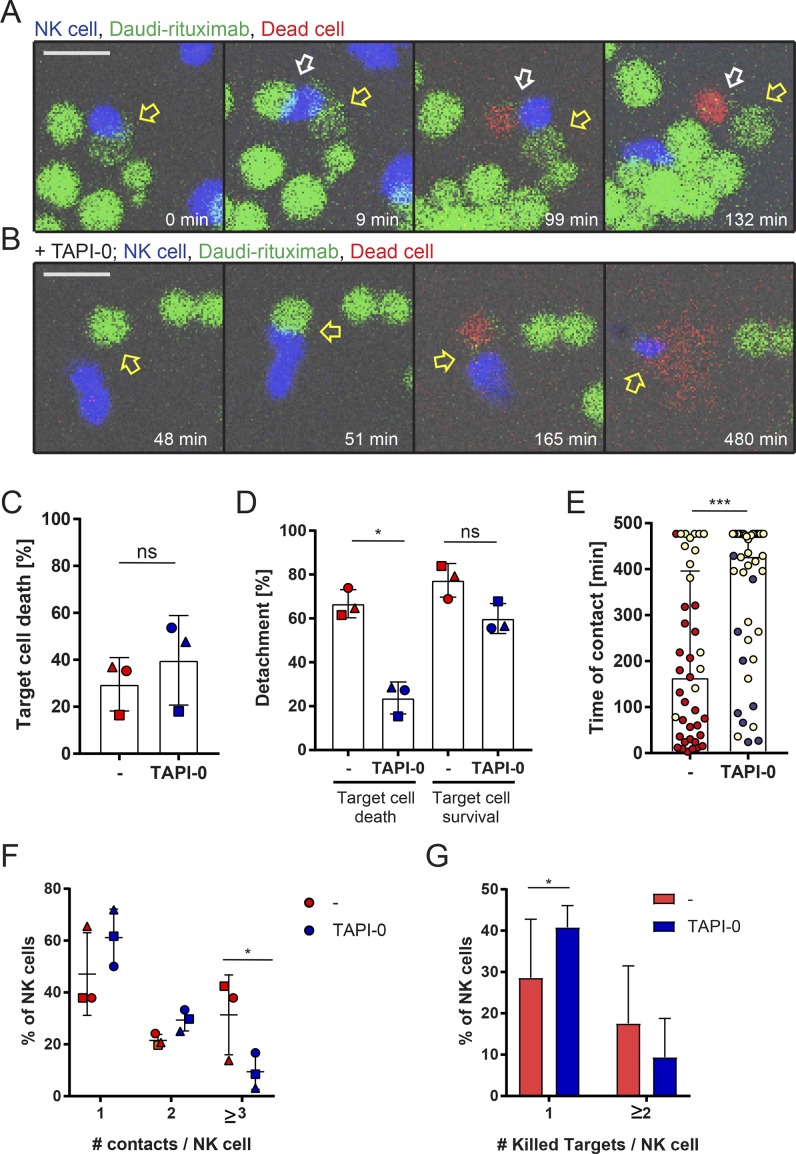
**In a 3D environment, CD16 shedding allows more interactions with target cells. (A–G)** NK cells and Daudi-rituximab were resuspended in Matrigel at E:T = 1:3 ± TAPI-0. Cell interactions were recorded every 3 min for 8 h. **(A and B)** Representative time-lapse–enlarged regions showing a composite of fluorescence and brightfield images. NK cells (blue), Daudi-rituximab (green), and dead cells (red) are shown at times indicated. Bars, 20 µm. **(A)** An NK cell (blue) formed a conjugate with a target (green; 0 min; yellow arrow). The NK cell formed a contact with another target (9 min; white arrow). The second target cell was killed (99 min), whereas the first one remained intact. The NK cell detached from both targets and moved to new target cells (132 min). **(B)** In the presence of TAPI-0, an NK cell established contact with a target cell (51 min; yellow arrow) and killed it (165 min). The NK cell remained attached to dead target until the end of acquisition (480 min). **(C)** Percentage of interactions that resulted in target cell lysis. **(D)** Percentage of NK cells that detached from target cells whether or not target cell was lysed. **(E)** Duration of all cytolytic NK cell–target cell contacts. Each point represents an individual contact. Yellow points indicate contacts still intact at the end of the acquisition (480 min). These points underestimate true contact times (median ± IQR). **(F)** Percentage of NK cells interacting with one, two, three, or more target cells. **(G)** Percentage of NK cells killing one or more target cells. Mean ± SD; symbols indicate different donors. *n* = 392 interactions from three independent experiments. *, P < 0.05; ***, P < 0.001 calculated by Student’s *t* test (C), one-way ANOVA (D), Mann-Whitney test (E), or two-way ANOVA (F and G).

It has been reported that the duration of immune synapses are different in a 3D environment (Gunzer et al., 2000), and thus we next set out to investigate whether CD16 shedding allows more interactions between cells in a 3D environment (formed by Matrigel; Fig. 9, A and B). Similar to microwells, levels of lysis were not significantly changed when CD16 shedding was inhibited (Fig. 9 C), but NK cell detachment was significantly affected (Fig. 9 D). The median contact time increased to 264 min (Fig. 9 E). In addition, within Matrigel, significantly fewer NK cells interacted with three or more targets when CD16 shedding was inhibited (Fig. 9 F). Moreover, when CD16 shedding was inhibited, NK cells more frequently killed a single target (Fig. 9 G). These data further indicate that efficient CD16 shedding leads to more interactions between NK cells and target cells, which in turn may impact NK cell serial killing.

## Discussion

Serial engagement of target cells is an important characteristic of NK cell immune surveillance. The relatively small number of NK cells in comparison with the number of potential target cells necessitates that each NK cell surveys and kills more than one target cell for efficient immunity. Indeed, the bulk of NK cell–mediated killing occurs in a sequential manner (Choi and Mitchison, 2013; Vanherberghen et al., 2013). However, factors controlling disassembly of the immune synapse, which thereby regulate the ability of NK cells to engage multiple target cells sequentially, have not been identified.

Tumor cells display a heterogeneous array of NK cell ligands, and how efficiently NK cells kill multiple target cells in such a complex environment has also been little studied. In this study, we demonstrate that the order in which target cells expressing different ligands are met affects the strength of NK cell cytolytic responses. When NK cells were repeatedly activated by rituximab, perforin secretion decreased but was restored to its initial level upon subsequent activation by MICA. Repeated stimulation of NK cells via MICA also decreased degranulation, but in this study, secretion could not be rescued by a subsequent stimulation with rituximab. We found that the mechanism underlying these differential outcomes involves shedding of CD16, which occurs upon NK cell activation through both CD16 and NKG2D. Shedding of CD16 renders the cells less sensitive to activation via that receptor, but they remain competent for further activation through NKG2D. Live-cell imaging established that lysis of Daudi-rituximab was reduced when NK cells had encountered Daudi-MICA first, but killing of Daudi-MICA was not affected by Daudi-rituximab as an initial contact. Together, these data establish that ligands on target cells can have a major impact on the sequential responsiveness of NK cells.

It is well established that cytotoxic cells assemble an immune synapse in order to execute target cell lysis. In this study, we demonstrated that cells formed stable cytolytic synapses quickly upon landing on MICA, but surprisingly, they remained motile on rituximab. Importantly, however, the inability of cells to form a static synapse on rituximab did not prevent their degranulation. Patterns of captured perforin further showed that cells displayed different dynamics on rituximab and MICA. On rituximab, perforin was secreted in elongated patches, whereas on MICA, it was secreted in closely arranged puncta. Inhibiting CD16 shedding and thus inhibiting NK cell motility on rituximab did not increase their degranulation. We propose in this study that the CD16-mediated NK cell contact interface is best described as a cytolytic kinapse, the term previously used to described a motile T cell–antigen-presenting cell (APC) contact, leading to signaling (Sims et al., 2007; Dustin, 2008; Choi and Mitchison, 2013).

Studying the individual interactions between NK cells and opsonized target cells revealed shedding of CD16 receptor as a major factor in NK cell detachment. Inhibition of CD16 cleavage resulted in impaired detachment irrespective of whether the target cell was lysed. In a model 3D environment, efficient detachment of NK cells from target cells increased subsequent interactions. It has been previously reported that the removal of surface receptor, mainly through internalization, can limit immune cell activation (Davis and Dustin, 2004; Dustin and Choudhuri, 2016). In line with this, we found that inhibition of CD16 shedding with TAPI-0 leads to higher secretion of IFN-γ, indicating that NK cells may still receive ongoing activating signals to secrete more cytokines even if the target cell has already been lysed. Importantly, NK cells that failed to detach were more likely to undergo apoptosis. This suggests that the detachment is a crucial step in preventing activation-induced death of NK cells.

Beyond the specific example of CD16, it is possible that cleavage of surface proteins represents a major mechanism for detachment of immune cells in a variety of other situations. ADAM17 can also cleave multiple NK cell ligands expressed by target cells, including MICA and ULBP2 (López-Cobo et al., 2016). This leads us to speculate that for some target cell–immune cell interactions, ADAM17 might also be involved in triggering disassembly of the synapse by cleavage of target cell ligands as well as, or instead of, cleavage of the immune cell receptors.

There have been several attempts to use inhibitors of ADAM17 therapeutically, but none have proved successful so far, consistent with the idea that some level of CD16 shedding is essential for optimal NK cell function. Our data indicate that rather than inhibiting CD16 shedding, it might be more beneficial for medical intervention to be able to boost CD16 reexpression upon its cleavage to augment NK cell responses.

In summary, we have shown in this study that shedding of CD16 has an unexpectedly complex impact on NK cell responses. Shedding of this receptor renders NK cells less potent at CD16-mediated activation as expected, but it promotes the detachment from opsonized targets to aid sequential target cell surveillance. Unzipping the NK cell immune synapse can lead to efficient serial engagement of multiple target cells and serves to sustain NK cell viability.

## Materials and methods

### Isolation of human primary NK cells

All experiments were performed using human primary NK cells except those presented in Figs. 3 and 8. Peripheral blood was acquired from the National Health Service blood service under ethics license REC 05/Q0401/108 (University of Manchester, Manchester, UK). Peripheral blood mononuclear cells were purified by density gradient centrifugation (Ficoll-Paque Plus; Amersham Pharmacia Biotech). Primary human NK cells were isolated using negative magnetic selection (Miltenyi Biotec) and cultured at 10^6^ cells/ml in clone media (DMEM, 30% Ham’s F-12, 10% human serum, 1 mM sodium pyruvate, 1% MEM nonessential amino acids [Sigma-Aldrich], 2 mM L-glutamine, 50 U/ml penicillin, 50 µg/ml streptomycin, and 50 µM β-mercaptoethanol [Gibco]) supplemented with 200 U/ml rhIL-2 (Roche) at 37°C and 5% CO_2_. NK cells were used 6 d after IL-2 stimulation.

### Cell lines

All cells were cultured at 37°C and 5% CO_2_. Daudi, Daudi-MICA, 221, 221-MICA, P815, and adherent Phoenix-Ampho cells were maintained in RPMI 1640 medium (Sigma-Aldrich) supplemented with 10% FBS, 2 mM L-glutamine, and 1% penicillin/streptomycin (Gibco). NK92 was cultured in α minimum MEM supplemented with 0.2 mM myoinositol, 0.02 mM folic acid, 2 mM L-glutamine, 1% MEM nonessential amino acids, 100 U/ml penicillin, 100 µg/ml streptomycin (Sigma-Aldrich), 12.5% FBS, 12.5% horse serum, and 0.1 mM, β-mercaptoethanol (Gibco). NK92 cell lines created by retroviral transduction were maintained in the presence of 20 µg/ml of the selecting antibiotic blasticidin (InvivoGen). All cell lines were routinely tested for mycoplasma infection using a PCR-based kit (PromoCell).

### Plasmid generation

The coding sequence of CD16 was ligated into a retroviral transfer vector pIB2. Noncleavable CD16-S197P was designed by introducing a point mutation by site-directed mutagenesis (Q5 site-directed mutagenesis kit; New England BioLabs, Inc.) using the forward primer 5′-TTTGGCAGTGcCAACCATCTC-3′ and reverse primer 5′-CCTTGAGTGATGGTGATG-3′ (New England BioLabs, Inc./Sigma-Aldrich; Jing et al., 2015). The presence of the point mutation was confirmed by DNA sequencing (GATC Biotech).

### Retroviral transduction of NK92/CD16 cells

The packaging cell line Phoenix-Ampho (ATCC) was transfected with lipofectamine LTX (Invitrogen) with a retroviral transfer vector pIB2 encoding WT CD16 or CD16-S197P. Viral supernatant was collected 72 h after transfection, and polybrene (Invitrogen) was added. The solution was filtered and used for centrifugations (400 *g* for 2 h at 32°C) for infection of 2 × 10^6^ cells. Cells expressing desired receptors were selected with 10 µg/ml of blasticidin (InvivoGen) in culture media.

### Preparation of coated slides

Preparation of eight-chambered glass coverslips was adapted from published protocols (Culley et al., 2009). Briefly, slides (1.5 Lab-Tek II; Nunc) were coated with 0.01% poly-L-lysine, dried, and coated with ligands for NK cells in PBS overnight at 4°C. 2.5 µg/ml MICA (Fc tagged; R&D Systems), 2.5 µg/ml MICA (His-tagged; Acro Biosystems), 10 µg/ml rituximab (GlaxoSmithKline), 2.5 µg/ml ULBP2 (Fc tagged; Abcam), 10 µg/ml anti-CD16 (clone 3G8; BioLegend), or 10 µg/ml anti-NKp30 mAb (clone P30-15; BioLegend), all with 2.5 µg/ml ICAM-1 (not tagged; R&D Systems) or 2.5 µg/ml Noggin (Fc tagged; R&D Systems), were used unless indicated otherwise. Where noted, 2.5 µg/ml ICAM-1 alone or 2.5 µg/ml of Noggin alone were also used as controls. In all experiments, recombinant human proteins MICA, ULBP2, and Noggin were used as Fc constructs and are referred throughout as MICA, ULBP2, and Noggin unless indicated otherwise. Slides were then washed with PBS and used for sample preparation.

### Perforin capture assay

Alongside NK cell ligands, 5 µg/ml anti-perforin mAb (clone CE2.10; Abcam) was coated on coverslips. Primary NK cells or transfected NK92 cells were incubated at 10^6^ cells/ml on coated slides for 1 h (unless indicated otherwise) at 37°C and 5% CO_2_. Then, supernatants were removed, and cells interacting with the surface were gently detached using nonenzymatic cell dissociation solution (Sigma-Aldrich) for 15 min at 37°C. NK cells were washed and used for sequential stimulation on fresh surfaces or stained to assess their surface receptor expression. Surfaces were washed three times with PBS and then blocked for 15 min with 1% BSA/PBS. Captured perforin was stained with Alexa Fluor 488–labeled anti-perforin mAb (clone dG9; BioLegend) overnight at 4°C. Slides were then washed and imaged by an inverted confocal microscope (TCS SP8; Leica Microsystems) using a 100× 1.4 NA oil-immersion objective. Images were analyzed using ImageJ software (National Institutes of Health).

### ELISA

Primary NK cells were incubated on polystyrene flat-bottomed 96-well plate (Nunc) coated with MICA (R&D Systems) or rituximab (GlaxoSmithKline), both with ICAM-1 (R&D Systems) or ICAM-1 alone in binding buffer (carbonate bicarbonate; Sigma-Aldrich) at 37°C for 18 h. Cell supernatants were collected and centrifuged at 350 *g* for 10 min at 4°C to remove cell debris. IFN-γ secretion was quantified from the supernatants by sandwich ELISA. For this, ELISA plates were coated with anti–IFN-γ mAb (1 µg/ml; clone NIB42; BD) in binding buffer (carbonate bicarbonate; Sigma-Aldrich) and blocked with 1% BSA/0.05% Tween-20/PBS. Supernatants were added to the plate and incubated for 1 h at RT. Plates were washed and incubated with biotinylated anti–IFN-γ mAb (1 µg/ml; clone 4S.B3; BD) and then streptavidin HRP (BD). The plates were developed with TMB ELISA substrate (Sigma-Aldrich), and the reaction was stopped with 1 N H_2_SO_4_. Absorbance was measured at 450 nm using a 570-nm reference line to compensate for optical interference.

### Flow cytometry

To assess surface expression of CD16 and NKG2D, cells were washed in 1% FBS/PBS (washing buffer) and blocked with 2% AB human serum/PBS (Sigma-Aldrich) for 10 min at 4°C and stained with viability dye (Zombie Aqua; BioLegend), BV421-labeled anti-CD56 mAb (clone HCD56; BioLegend), Alexa Fluor 647–labeled anti-CD16 mAb (clone 3G8; BioLegend), phycoerythrin (PE)-labeled anti-NKG2D mAb (clone 1D11; BioLegend), or isotype-matched control mAbs (mouse IgG1 isotype control conjugated with BV421, Alexa Fluor 647, or PE; clone MOPC-21; BioLegend) for 30 min at 4°C. For surface levels of MICA, cells were stained with APC-labeled anti-MICA mAb (159227; R&D Systems). Opsonized rituximab was labeled with FITC-labeled anti–human IgG (Fc-specific) mAb (Sigma-Aldrich). For the assessment of surface receptor expression levels, only live singlets were considered. To assess sequential killing of target cells, P815, Daudi-MICA, or Daudi-rituximab were incubated for 1.5 h with NK cells at effector/target cell ratio (E:T) of 1:1. After this, Daudi-MICA or Daudi-rituximab labeled according to manufacturer’s instructions (Violet cell trace proliferation kit; Thermo Fisher Scientific) were added for another 1.5 h. Samples were incubated in the presence of 2 µM caspase 3/7 green stain (Thermo Fisher Scientific) at 37°C. Cells were then washed, blocked with 2% human AB serum/PBS, and stained with Alexa Fluor 647–labeled anti-CD16 mAb (clone 3G8; BioLegend) for 30 min at 4°C. Finally, cells were washed, fixed in 2% PFA/PBS, assessed by flow cytometry (FACS Canto II flow cytometer; BD), and analyzed (FlowJo_V10 software).

### CD107a degranulation assay

Primary NK cells were incubated on coated surfaces as indicated in the presence of brefeldin (GolgiPlug; 1:1,000 dilution; BD), monensin (GolgiSTOP; 1:1,000 dilution; BD), and Alexa Fluor 647–labeled anti–LAMP-1 mAb (clone H4A3; Santa Cruz Biotechnology) or isotype-matched control mAb (mouse IgG1 isotype control conjugated with Alexa Fluor 647; clone MOPC-21; BioLegend) for 4 h at 37°C and 5% CO_2_. After incubation, cells were washed and stained with dead cell marker (Zombie Aqua viability dye), Brilliant Violet 421–labeled anti-CD56 mAb (clone HCD56; BioLegend), and Alexa Fluor 647–labeled anti–LAMP-1 mAb or isotype-matched control mAb. Finally, cells were washed in 1% FBS/PBS, fixed in 2% PFA/PBS, assessed by flow cytometry (FACS Canto II flow cytometer), and analyzed (FlowJo_V10 software).

### F-actin analysis

For F-actin analysis, cells were allowed to settle on coated slides for 5 min (unless indicated otherwise) at 37°C, fixed in 4% PFA/PBS at RT for 20 min, and permeabilized with 0.1% Triton X-100/PBS at RT for 10 min. F-actin was stained with Alexa Fluor 488–labeled phalloidin (1:200 dilution in PBS; Invitrogen) and imaged by confocal microscopy (TCS SP8) with a 100× 1.4 NA oil-immersion objective. Images were exported to ImageJ, and the percentage of cells with different distributions of F-actin was scored.

### IRM imaging

Primary NK cells were incubated on chambered glass coverslips (1.5 Lab-Tek II), coated with rituximab or MICA, both with ICAM-1 or ICAM-1 alone. Live imaging of individual NK cells landing on coated surfaces was performed by confocal microscopy (TCS SP8) at 37°C and 5% CO_2_. 560 nm excitation at a low laser power and signal detection on a photomultiplier tube detector set to 555–570-nm detection wavelengths on reflection mode were used. IRM images were recorded at rate of one frame per second. For analysis, stacks were reduced 1:5 using the Stacks plugin in ImageJ. Stacks were smoothed and thresholded using Otsu method. Spreading area and circularity were analyzed using Analyze Particles plugin for ImageJ.

### Migration assay

First, primary NK cells were stained with 1 µM Calcein Red-Orange (Thermo Fisher Scientific), and then labeled cells were incubated at 5 × 10^5^ cells/ml into coated chambered glass coverslips in clone media. Where indicated, 1 µM TAPI-0 was added to wells before imaging. Time-lapse imaging was performed at 37°C and 5% CO_2_ immediately after cells landed on slides at rate of one frame per 30 s for 45 min (TCS SP8). Acquired fluorescent, brightfield, and IRM images were merged, and individual cells were manually tracked using MATLAB-based Cell Tracker software (Piccinini et al., 2016).

### Time-lapse imaging in microwells or in 3D Matrigel

Microwells were coated with 10 µg/ml fibronectin (Sigma-Aldrich) and blocked with clone media. Primary NK cells or NK92 transfectants were stained with 1 µM Calcein Red-Orange or 5 µM Cell Trace Violet proliferation kit, Daudi cells were labeled with 0.3 µM Calcein Green or 1 µM Calcein Red-Orange, and Daudi-MICA was labeled with 0.3 µM Calcein Green. To opsonize Daudi cells, labeled cells were incubated in the presence of 10 µg/ml rituximab for 1 h at 37°C. Labeled NK cells were then added to Daudi-rituximab at E:T = 1:3 or to Daudi-rituximab and Daudi-MICA at E:T:T = 1:1.5:1.5 as indicated in clone media supplemented with 1 µl ToPro-3 to discriminate dead cells (Thermo Fisher Scientific). For imaging in microwells, cells were mixed and immediately placed into microchips with wells of dimensions 450 × 450 × 300 µm^3^ (large wells) or 50 × 50 × 300 µm^3^ (small wells). For imaging in Matrigel, cells were resuspended in a cold Matrigel solution diluted 1:4 with clone media and added to chambered glass coverslips. Time-lapse imaging was performed using an inverted confocal microscope with 20× 0.75 NA objective (SP8; Leica Microsystems) at 37°C and 5% CO_2_ for 8 h, with an image acquired every 3 min. Where indicated, 1 µM TAPI-0 (Santa Cruz Biotechnology) was added to cells before acquisition. Wells with or without TAPI-0 and wells with NK92/CD16-WT or NK92/CD16-S197P were imaged in parallel by using two separate basins, each covering multiple wells of the microchip. Images were analyzed using ImageJ.

### Quantification and statistical analysis

Sample sizes chosen were appropriate to provide enough power for statistical tests used in this study. For each dataset, a D’Agostino and Pearson omnibus test or Shapiro-Wilk normality test were used to evaluate the distribution of obtained values. The statistical significance of differences between two groups of data with normal Gaussian distribution was examined using a two-tailed Student's *t* test. The statistical significance between three or more conditions was assessed by one-way ANOVA or two-way ANOVA.

If at least one of the conditions did not have a normal distribution, nonparametric tests were used. A Mann-Whitney test was used to compare two groups of data, and multiple comparisons were evaluated using a Kruskal-Wallis test or matched-values Friedman test with Dunn’s post testing. Differences were defined as nonsignificant where P ≥ 0.05 and were statistically significant where *, P < 0.05; **, P < 0.01; ***, P < 0.001; and ****, P < 0.0001. All statistical analyses were performed using Prism (7.0; GraphPad Software).

### Online supplemental material

Fig. S1 shows a microscopy-based assay to assess perforin secretion on a single-cell level. Fig. S2 shows how the concentration of activating ligands affects NK cell degranulation. Fig. S3 shows how serial responses are independent of the type of ligand used. Fig. S4 shows how surface expression of CD16 and NKG2D are differentially affected by the ligands expressed by target cells. Fig. S5 shows the effects of ligand concentration and TAPI-0 on various NK cell activation responses. Video 1 shows NK cell interactions with rituximab-coated surfaces. Video 2 shows NK cell interactions with MICA-coated surfaces. Video 3 shows how an NK cell detaches from an opsonized target cell. Video 4 shows how TAPI-0 prevents the detachment from an opsonized target cell.

## Supplementary Material

Supplemental Materials (PDF)

Video 1

Video 2

Video 3

Video 4

## References

[bib1] Bhat R., and Watzl C. 2007 Serial killing of tumor cells by human natural killer cells--enhancement by therapeutic antibodies. PLoS One. 2:e326 10.1371/journal.pone.0000326 17389917 PMC1828617

[bib2] Bi J., and Tian Z. 2017 NK Cell Exhaustion. Front. Immunol. 8:760 10.3389/fimmu.2017.00760 28702032 PMC5487399

[bib3] Carisey A.F., Mace E.M., Saeed M.B., Davis D.M., and Orange J.S. 2018 Nanoscale Dynamism of Actin Enables Secretory Function in Cytolytic Cells. Curr. Biol. 28:489–502. 29398219 10.1016/j.cub.2017.12.044 PMC5835143

[bib4] Cartwright A.N., Griggs J., and Davis D.M. 2014 The immune synapse clears and excludes molecules above a size threshold. Nat. Commun. 5:5479 10.1038/ncomms6479 25407222 PMC4248232

[bib5] Cerboni C., Ardolino M., Santoni A., and Zingoni A. 2009 Detuning CD8+ T lymphocytes by down-regulation of the activating receptor NKG2D: role of NKG2D ligands released by activated T cells. Blood. 113:2955–2964. 10.1182/blood-2008-06-165944 19124832

[bib6] Cheson B.D., and Leonard J.P. 2008 Monoclonal antibody therapy for B-cell non-Hodgkin’s lymphoma. N. Engl. J. Med. 359:613–626. 10.1056/NEJMra0708875 18687642

[bib7] Choi P.J., and Mitchison T.J. 2013 Imaging burst kinetics and spatial coordination during serial killing by single natural killer cells. Proc. Natl. Acad. Sci. USA. 110:6488–6493. 10.1073/pnas.1221312110 23576740 PMC3631668

[bib8] Clynes R.A., Towers T.L., Presta L.G., and Ravetch J.V. 2000 Inhibitory Fc receptors modulate in vivo cytotoxicity against tumor targets. Nat. Med. 6:443–446. 10.1038/74704 10742152

[bib9] Costello R.T., Sivori S., Marcenaro E., Lafage-Pochitaloff M., Mozziconacci M.-J., Reviron D., Gastaut J.-A., Pende D., Olive D., and Moretta A. 2002 Defective expression and function of natural killer cell-triggering receptors in patients with acute myeloid leukemia. Blood. 99:3661–3667. 10.1182/blood.V99.10.3661 11986221

[bib10] Coudert J.D., Zimmer J., Tomasello E., Cebecauer M., Colonna M., Vivier E., and Held W. 2005 Altered NKG2D function in NK cells induced by chronic exposure to NKG2D ligand-expressing tumor cells. Blood. 106:1711–1717. 10.1182/blood-2005-03-0918 15886320

[bib11] Culley F.J., Johnson M., Evans J.H., Kumar S., Crilly R., Casasbuenas J., Schnyder T., Mehrabi M., Deonarain M.P., Ushakov D.S., 2009 Natural killer cell signal integration balances synapse symmetry and migration. PLoS Biol. 7:e1000159 10.1371/journal.pbio.1000159 19636352 PMC2707003

[bib12] Davenport A.J., Cross R.S., Watson K.A., Liao Y., Shi W., Prince H.M., Beavis P.A., Trapani J.A., Kershaw M.H., Ritchie D.S., 2018 Chimeric antigen receptor T cells form nonclassical and potent immune synapses driving rapid cytotoxicity. Proc. Natl. Acad. Sci. USA. 115:E2068–E2076. 10.1073/pnas.1716266115 29440406 PMC5834689

[bib13] Davis D.M., and Dustin M.L. 2004 What is the importance of the immunological synapse? Trends Immunol. 25:323–327. 10.1016/j.it.2004.03.007 15145322

[bib14] Davis D.M., Chiu I., Fassett M., Cohen G.B., Mandelboim O., and Strominger J.L. 1999 The human natural killer cell immune synapse. Proc. Natl. Acad. Sci. USA. 96:15062–15067. 10.1073/pnas.96.26.15062 10611338 PMC24773

[bib15] Dustin M.L. 2008 T-cell activation through immunological synapses and kinapses. Immunol. Rev. 221:77–89. 10.1111/j.1600-065X.2008.00589.x 18275476

[bib16] Dustin M.L., and Choudhuri K. 2016 Signaling and Polarized Communication Across the T Cell Immunological Synapse. Annu. Rev. Cell Dev. Biol. 32:303–325. 10.1146/annurev-cellbio-100814-125330 27501450

[bib17] Edwards J.C., Szczepanski L., Szechinski J., Filipowicz-Sosnowska A., Emery P., Close D.R., Stevens R.M., and Shaw T. 2004 Efficacy of B-cell-targeted therapy with rituximab in patients with rheumatoid arthritis. N. Engl. J. Med. 350:2572–2581. 10.1056/NEJMoa032534 15201414

[bib18] Fauriat C., Long E.O., Ljunggren H.-G., and Bryceson Y.T. 2010 Regulation of human NK-cell cytokine and chemokine production by target cell recognition. Blood. 115:2167–2176. 10.1182/blood-2009-08-238469 19965656 PMC2844017

[bib19] Goodier M.R., Lusa C., Sherratt S., Rodriguez-Galan A., Behrens R., and Riley E.M. 2016 Sustained Immune Complex-Mediated Reduction in CD16 Expression after Vaccination Regulates NK Cell Function. Front. Immunol. 7:384 10.3389/fimmu.2016.00384 27725819 PMC5035824

[bib20] Groh V., Wu J., Yee C., and Spies T. 2002 Tumour-derived soluble MIC ligands impair expression of NKG2D and T-cell activation. Nature. 419:734–738. 10.1038/nature01112 12384702

[bib21] Guillerey C., Huntington N.D., and Smyth M.J. 2016 Targeting natural killer cells in cancer immunotherapy. Nat. Immunol. 17:1025–1036. 10.1038/ni.3518 27540992

[bib22] Guldevall K., Vanherberghen B., Frisk T., Hurtig J., Christakou A.E., Manneberg O., Lindström S., Andersson-Svahn H., Wiklund M., and Önfelt B. 2010 Imaging immune surveillance of individual natural killer cells confined in microwell arrays. PLoS One. 5:e15453 10.1371/journal.pone.0015453 21103395 PMC2980494

[bib23] Gunzer M., Schäfer A., Borgmann S., Grabbe S., Zänker K.S., Bröcker E.B., Kämpgen E., and Friedl P. 2000 Antigen presentation in extracellular matrix: interactions of T cells with dendritic cells are dynamic, short lived, and sequential. Immunity. 13:323–332. 10.1016/S1074-7613(00)00032-7 11021530

[bib24] Gwalani L.A., and Orange J.S. 2018 Single Degranulations in NK Cells Can Mediate Target Cell Killing. J. Immunol. 200:3231–3243. 10.4049/jimmunol.1701500 29592963 PMC6020067

[bib25] Halle S., Keyser K.A., Stahl F.R., Busche A., Marquardt A., Zheng X., Galla M., Heissmeyer V., Heller K., Boelter J., 2016 In vivo killing capacity of cytotoxic T cells is limited and involves dynamic interactions and T cell cooperativity. Immunity. 44:233–245. 10.1016/j.immuni.2016.01.010 26872694 PMC4846978

[bib26] Hsu H.-T., Mace E.M., Carisey A.F., Viswanath D.I., Christakou A.E., Wiklund M., Önfelt B., and Orange J.S. 2016 NK cells converge lytic granules to promote cytotoxicity and prevent bystander killing. J. Cell Biol. 215:875–889. 27903610 10.1083/jcb.201604136 PMC5166499

[bib27] Jenkins M.R., Rudd-Schmidt J.A., Lopez J.A., Ramsbottom K.M., Mannering S.I., Andrews D.M., Voskoboinik I., and Trapani J.A. 2015 Failed CTL/NK cell killing and cytokine hypersecretion are directly linked through prolonged synapse time. J. Exp. Med. 212:307–317. 10.1084/jem.20140964 25732304 PMC4354371

[bib28] Jing Y., Ni Z., Wu J., Higgins L., Markowski T.W., Kaufman D.S., and Walcheck B. 2015 Identification of an ADAM17 cleavage region in human CD16 (FcγRIII) and the engineering of a non-cleavable version of the receptor in NK cells. PLoS One. 10:e0121788 10.1371/journal.pone.0121788 25816339 PMC4376770

[bib29] Konjević G., Mirjacić Martinović K., Vuletić A., Jović V., Jurisić V., Babović N., and Spuzić I. 2007 Low expression of CD161 and NKG2D activating NK receptor is associated with impaired NK cell cytotoxicity in metastatic melanoma patients. Clin. Exp. Metastasis. 24:1–11. 10.1007/s10585-006-9043-9 17295095

[bib30] Ljunggren H.G., and Malmberg K.J. 2007 Prospects for the use of NK cells in immunotherapy of human cancer. Nat. Rev. Immunol. 7:329–339. 10.1038/nri2073 17438573

[bib31] López-Cobo S., Campos-Silva C., and Valés-Gómez M. 2016 Glycosyl-Phosphatidyl-Inositol (GPI)-Anchors and Metalloproteases: Their Roles in the Regulation of Exosome Composition and NKG2D-Mediated Immune Recognition. Front. Cell Dev. Biol. 4:97 10.3389/fcell.2016.00097 27672635 PMC5019032

[bib32] Martz E. 1976 Multiple target cell killing by the cytolytic T lymphocyte and the mechanism of cytotoxicity. Transplantation. 21:5–11. 10.1097/00007890-197601000-00002 1082188

[bib33] Molfetta R., Quatrini L., Zitti B., Capuano C., Galandrini R., Santoni A., and Paolini R. 2016 Regulation of NKG2D expression and signaling by endocytosis. Trends Immunol. 37:790–802. 10.1016/j.it.2016.08.015 27667711

[bib34] Netter P., Anft M., and Watzl C. 2017 Termination of the Activating NK Cell Immunological Synapse Is an Active and Regulated Process. J. Immunol. 199:2528–2535. 10.4049/jimmunol.1700394 28835459

[bib35] Ogasawara K., Hamerman J.A., Hsin H., Chikuma S., Bour-Jordan H., Chen T., Pertel T., Carnaud C., Bluestone J.A., and Lanier L.L. 2003 Impairment of NK cell function by NKG2D modulation in NOD mice. Immunity. 18:41–51. 10.1016/S1074-7613(02)00505-8 12530974

[bib36] Orange J.S. 2008 Formation and function of the lytic NK-cell immunological synapse. Nat. Rev. Immunol. 8:713–725. 10.1038/nri2381 19172692 PMC2772177

[bib37] Peruzzi G., Femnou L., Gil-Krzewska A., Borrego F., Weck J., Krzewski K., and Coligan J.E. 2013 Membrane-type 6 matrix metalloproteinase regulates the activation-induced downmodulation of CD16 in human primary NK cells. J. Immunol. 191:1883–1894. 10.4049/jimmunol.1300313 23851692 PMC3745217

[bib38] Piccinini F., Kiss A., and Horvath P. 2016 CellTracker (not only) for dummies. Bioinformatics. 32:955–957. 10.1093/bioinformatics/btv686 26589273

[bib39] Quatrini L., Molfetta R., Zitti B., Peruzzi G., Fionda C., Capuano C., Galandrini R., Cippitelli M., Santoni A., and Paolini R. 2015 Ubiquitin-dependent endocytosis of NKG2D-DAP10 receptor complexes activates signaling and functions in human NK cells. Sci. Signal. 8:ra108 10.1126/scisignal.aab2724 26508790

[bib40] Romee R., Foley B., Lenvik T., Wang Y., Zhang B., Ankarlo D., Luo X., Cooley S., Verneris M., Walcheck B., and Miller J. 2013 NK cell CD16 surface expression and function is regulated by a disintegrin and metalloprotease-17 (ADAM17). Blood. 121:3599–3608. 10.1182/blood-2012-04-425397 23487023 PMC3643761

[bib41] Sims T.N., Soos T.J., Xenias H.S., Dubin-Thaler B., Hofman J.M., Waite J.C., Cameron T.O., Thomas V.K., Varma R., Wiggins C.H., 2007 Opposing effects of PKCtheta and WASp on symmetry breaking and relocation of the immunological synapse. Cell. 129:773–785. 10.1016/j.cell.2007.03.037 17512410

[bib42] Vanherberghen B., Olofsson P.E., Forslund E., Sternberg-Simon M., Khorshidi M.A., Pacouret S., Guldevall K., Enqvist M., Malmberg K.-J., Mehr R., and Önfelt B. 2013 Classification of human natural killer cells based on migration behavior and cytotoxic response. Blood. 121:1326–1334. 10.1182/blood-2012-06-439851 23287857

[bib43] Varadarajan N., Julg B., Yamanaka Y.J., Chen H., Ogunniyi A.O., McAndrew E., Porter L.C., Piechocka-Trocha A., Hill B.J., Douek D.C., 2011 A high-throughput single-cell analysis of human CD8^+^ T cell functions reveals discordance for cytokine secretion and cytolysis. J. Clin. Invest. 121:4322–4331. 10.1172/JCI58653 21965332 PMC3204845

[bib44] Vivier E., Tomasello E., Baratin M., Walzer T., and Ugolini S. 2008 Functions of natural killer cells. Nat. Immunol. 9:503–510. 10.1038/ni1582 18425107

[bib45] Vivier E., Raulet D.H., Moretta A., Caligiuri M.A., Zitvogel L., Lanier L.L., Yokoyama W.M., and Ugolini S. 2011 Innate or adaptive immunity? The example of natural killer cells. Science. 331:44–49. 10.1126/science.1198687 21212348 PMC3089969

[bib46] Voskoboinik I., Whisstock J.C., and Trapani J.A. 2015 Perforin and granzymes: function, dysfunction and human pathology. Nat. Rev. Immunol. 15:388–400. 10.1038/nri3839 25998963

[bib47] Wiemann K., Mittrücker H.W., Feger U., Welte S.A., Yokoyama W.M., Spies T., Rammensee H.G., and Steinle A. 2005 Systemic NKG2D down-regulation impairs NK and CD8 T cell responses in vivo. J. Immunol. 175:720–729. 10.4049/jimmunol.175.2.720 16002667

