# Supplementary material for: Shedding of CD16 disassembles the NK cell immune synapse and boosts serial engagement of target cells
Source: J Cell Biol. 2018 Sep 3;217(9):3267–83. doi: 10.1083/jcb.201712085 (PMC6122987; doi:10.1083/jcb.201712085)
Supplement: Supplemental Materials (PDF) [file JCB_201712085_sm.pdf]

Supplemental material

Srpan et al., https://doi.org/10.1083/jcb.201712085

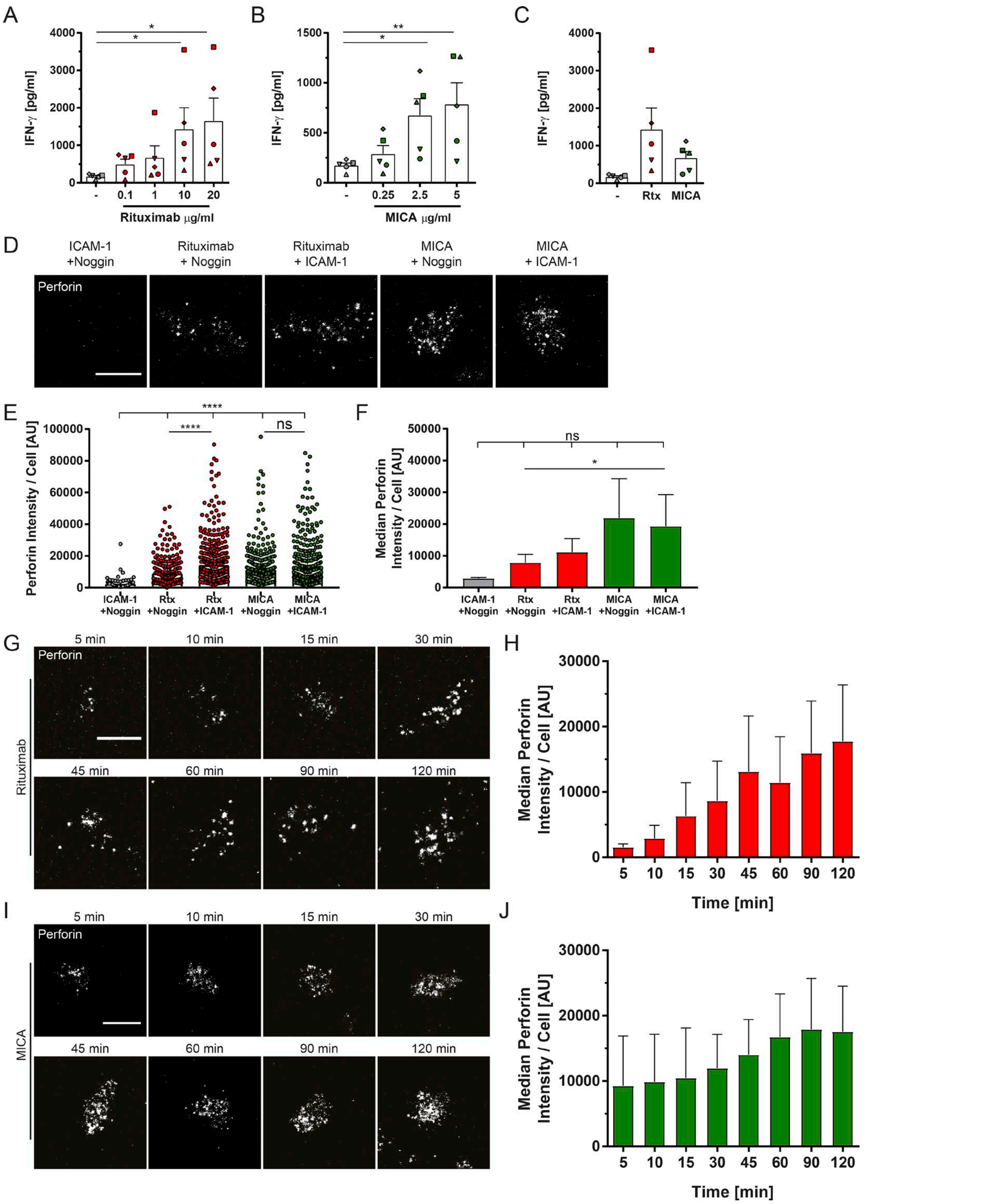

Figure S1. **A microscopy-based assay to assess perforin secretion on a single cell level. (A–C)** NK cells were stimulated for 18 h in a 96-well plate coated with varying concentrations of activating ligands (rituximab or MICA, both with ICAM-1 or ICAM-1 alone). The amount of IFN-γ secreted into the supernatant was measured by ELISA. n = 5; mean ± SEM; symbols represent different donors. **(D–F)** NK cells were incubated for 1 h on surfaces coated with activating ligands or corresponding negative controls as indicated. Secreted perforin was captured and stained with Alexa Fluor 488–labeled anti-perforin mAb after cells were removed and imaged by confocal microscopy. **(D)** Representative images of perforin secreted from one cell. Bar, 10 µm. **(E)** Quantitative analysis of perforin secreted by cells from a representative donor. Each point represents the fluorescence (IFI) from perforin captured from one cell (median ± IQR). **(F)** Median IFI values of perforin secretion from different donors (mean ± SEM). **(G–J)** NK cells were incubated on surfaces coated with rituximab or MICA and both with ICAM-1 for 5–120 min as indicated. Cells were then removed, and captured perforin was stained with Alexa Fluor 488–conjugated anti-perforin mAb and imaged by confocal microscopy. **(G and I)** Panels show representative images of perforin secreted from one cell at each time point. Bars, 10 µm. **(H and J)** Median IFI values of captured perforin (mean ± SEM). *, P < 0.05; **, P < 0.01; ***, P < 0.001; ****, P < 0.0001 calculated by one-way ANOVA (A–C), Kruskal-Wallis test (E), or Friedman test (F).

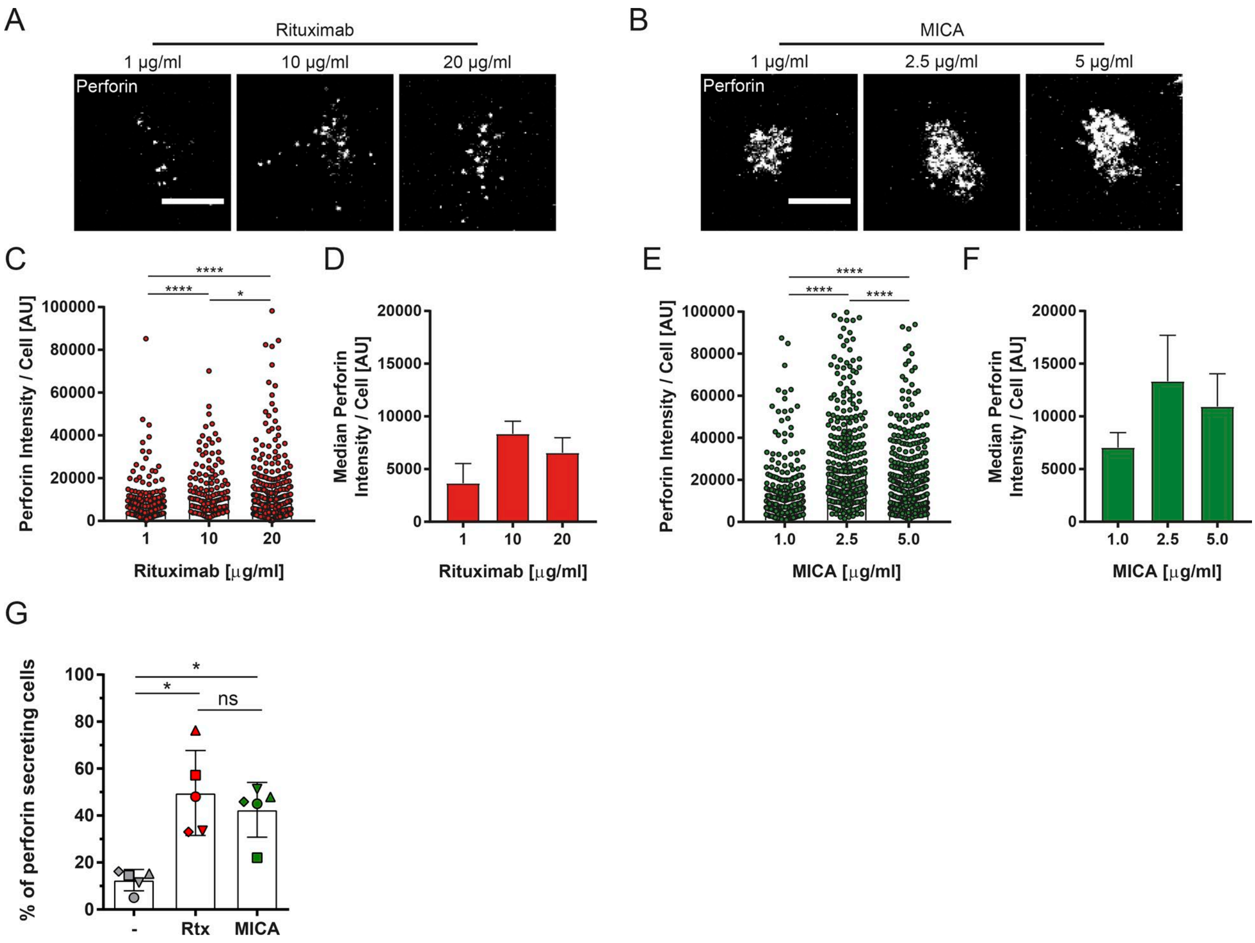

Figure S2. **Concentration of activating ligands affects the extent of NK cell degranulation. (A–F)** Glass slides were coated with different concentrations of rituximab (Rtx; 1, 10, or 20 µg/ml) or MICA (1.0, 2.5, or 5.0 µg/ml), both with ICAM-1. NK cells were incubated for 1 h, and secreted perforin was captured and stained with Alexa Fluor 488–conjugated anti-perforin mAb and imaged by confocal microscopy. **(A and B)** Representative images of perforin secreted per cell on various concentrations of activating ligands as indicated. Bars, 10 µm. **(C and E)** Quantitative analysis of perforin secreted by cells from a representative donor. Each point represents the fluorescence (IFI) from perforin captured from one cell (median ± IQR). **(D and F)** Median IFI values of perforin secretion from different donors (n = 3; mean ± SEM). **(G)** To estimate the percentage of perforin-secreting cells, two sets of coated surfaces (rituximab or MICA, both with ICAM-1, or ICAM-1 alone [–]) were used in parallel. The same number of cells was incubated in each well. One well was used for the perforin capture assay, while cells were fixed and stained with phalloidin in the parallel well. The proportion of perforin-secreting cells was calculated by dividing the number of perforin-secreting cells by the total number of cells in the field of view (n = 5; mean ± SD; symbols represent different donors). *, $P < 0.05$; ****, $P < 0.0001$ calculated by Kruskal-Wallis test (C and E) and one-way ANOVA (G).

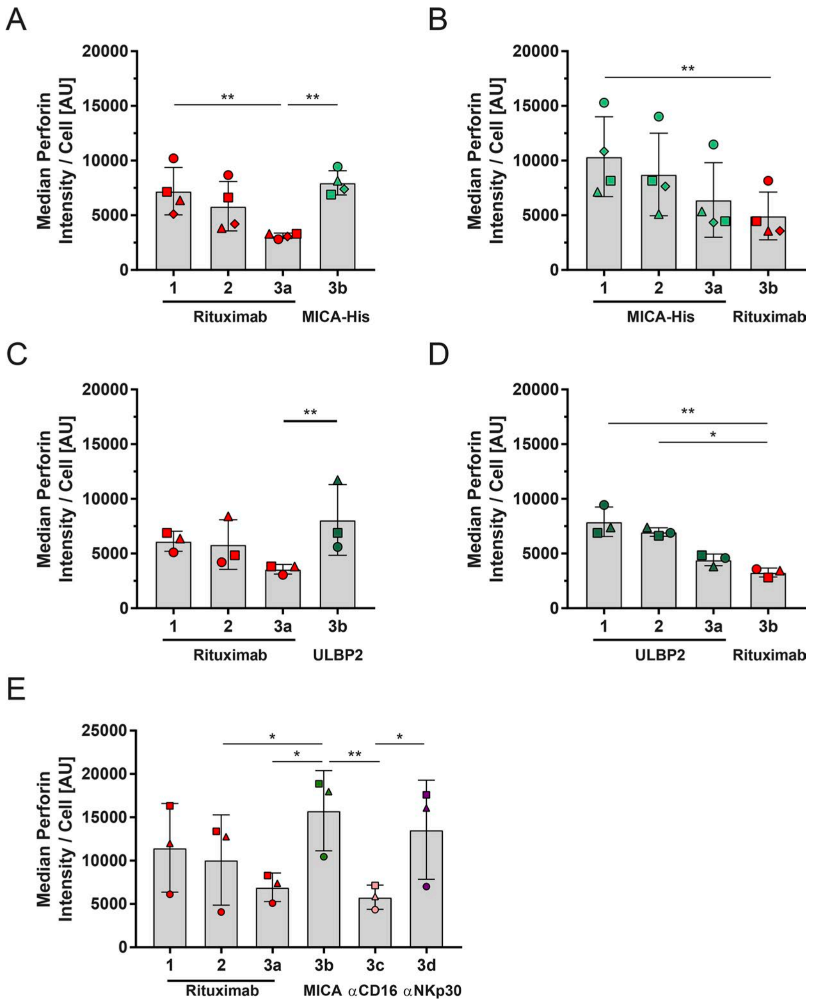

Figure S3. **Serial responses are independent of the type of the ligand used. (A–E)** NK cells were sequentially activated for 1 h through CD16, NKG2D, or NKp30 on surfaces coated with their cognate ligands (rituximab, anti-CD16 mAb, MICA, MICA-His, ULBP2, or anti-NKp30), all with ICAM-1 as indicated. After two sequential stimulations through the same ligand, cells were split into two (A–D) or three (E) groups. One group were stimulated through the same ligand, and the other were introduced to a different ligand as indicated. Secreted perforin was captured and stained with an Alexa Fluor 488–labeled anti-perforin mAb and imaged by confocal microscopy. Median fluorescence of perforin secretion from different donors; n = 4 (A and B) and n = 3 (C–E); mean ± SEM; symbols represent different donors. *, P < 0.05; **, P < 0.01 calculated by Friedman test.

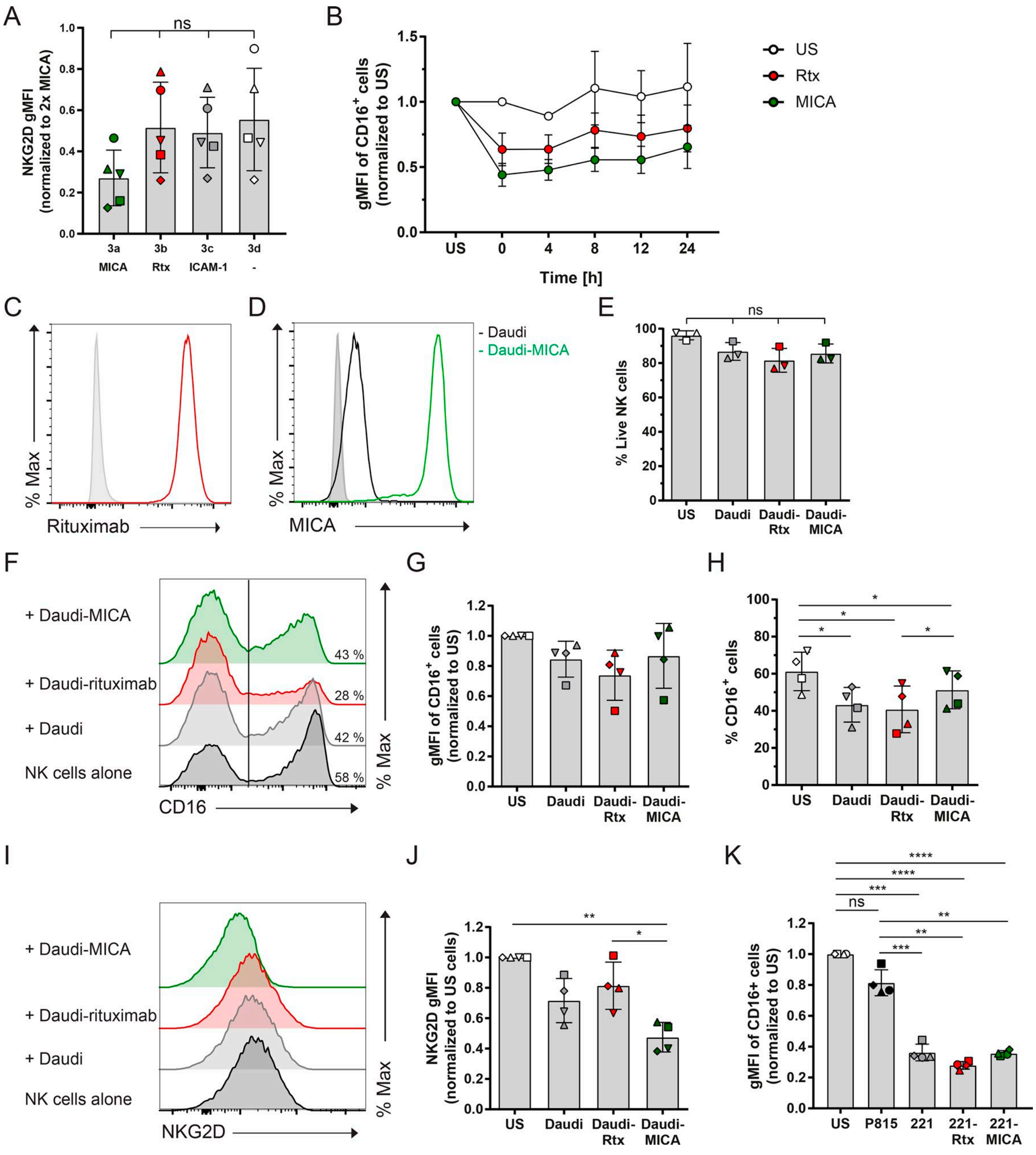

Figure S4. **Surface expression of CD16 and NKG2D are differentially affected by the ligands expressed by target cells. (A)** NK cells were sequentially stimulated on surface coated with MICA and ICAM-1 twice for 1 h. Then, cells were split into four groups, each incubated on a surface coated with MICA or rituximab (Rtx), both with ICAM-1, ICAM-1 alone, or an uncoated surface. After 1 h, cells were removed and stained for the surface expression of NKG2D. Plotted are gMFI values normalized to cells stimulated twice on MICA (n = 5; mean ± SD; symbols indicate different donors). **(B)** CD16 expression over 24 h after stimulation. NK cells were stimulated on surfaces coated with rituximab (red) or MICA (green), both with ICAM1 for 1 h. Then, cells were removed, washed, and incubated in clone media. The expression levels of CD16 were assessed by FACS at 0, 4, 12, and 24 h after stimulation and compared with CD16 on nonactivated NK cells (white). gMFI of CD16⁺ NK cells. n = 4; mean ± SEM. **(C)** Daudi cells were opsonized with rituximab for 1 h. Rituximab was stained with FITC-labeled anti-Fc mAb. Fc staining was compared with isotype-matched control (gray). **(D)** Daudi cells (black) and Daudi-MICA (green) were stained for surface MICA with APC-labeled anti-MICA mAb. Expression levels were compared with isotype-matched control mAb (gray). **(E–J)** NK cells were incubated with Daudi (gray), Daudi-rituximab (red), or Daudi-MICA (green) for 1.5 h. To distinguish the cells, target cells were labeled with cell trace dye. Expression levels of CD16 and NKG2D on NK cells were assessed by flow cytometry and compared with the expression on unstimulated (US) NK cells (black). n = 4 independent experiments; mean ± SD; symbols represent different donors. **(E)** Viability of NK cells after incubation with Daudi cells. **(F)** Representative histograms of CD16 upon incubation with target cells. Vertical line denotes cutoff for $CD16^+$ cells. Percentage of cells that were $CD16^+$ are shown from a representative donor. **(G)** CD16 gMFI of $CD16^+$ cells upon stimulation with target cells normalized to unstimulated cells. **(H)** Percentage of $CD16^+$ cells upon incubation with target cells. **(I)** Representative histograms of NKG2D expression upon incubation with target cells. **(J)** gMFI of NKG2D normalized to unstimulated cells. **(K)** NK cells were incubated with P815, 221, 221-rituximab, or 221-MICA for 1 h. All target cells were labeled with cell trace dye. After 1 h, surface levels of CD16 were assessed by flow cytometry. Panel shows CD16 gMFI of $CD16^+$ cells upon stimulation with target cells normalized to unstimulated cells. *, $P < 0.05$; **, $P < 0.01$; ***, $P < 0.001$; ****, $P < 0.0001$ calculated by one-way ANOVA.

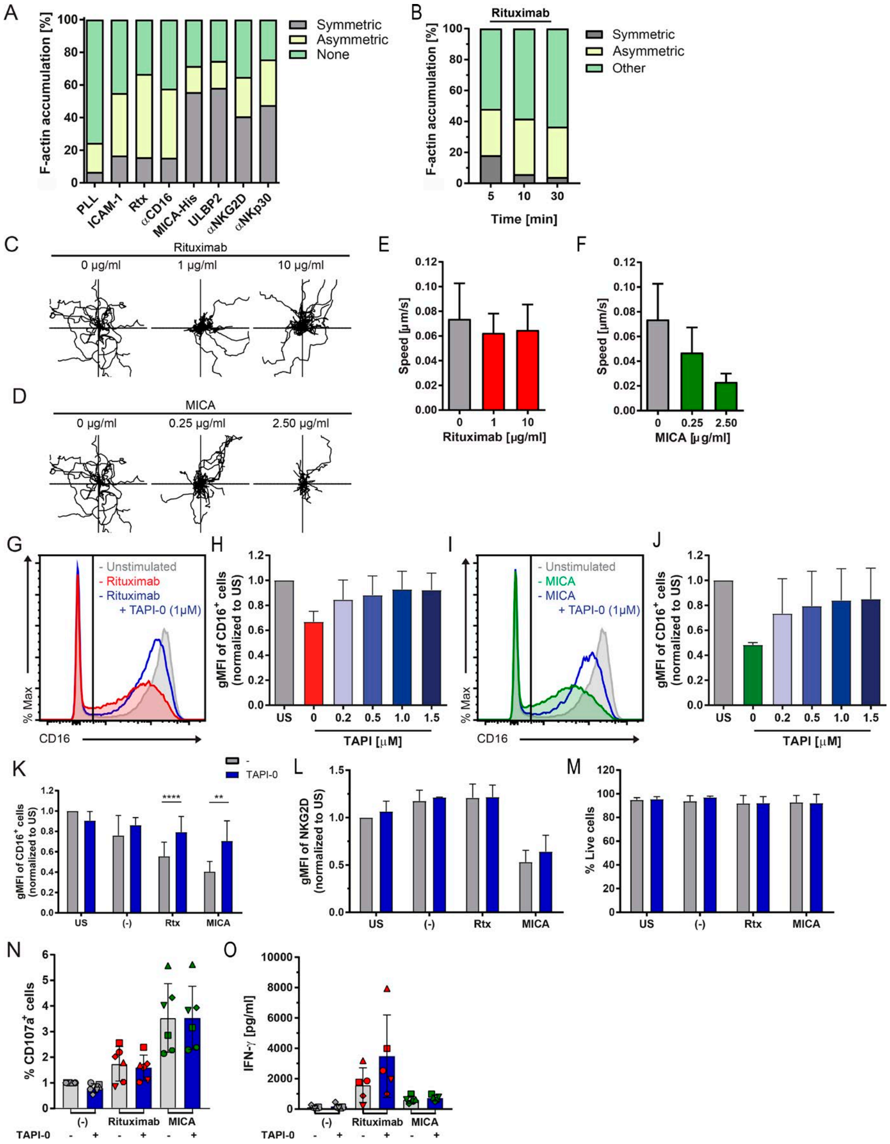

Figure S5. **The effects of ligand concentration and TAPI-0 on various NK cell activation responses.** **(A)** Cells were incubated on poly-L-lysine (PLL) or ICAM-1 alone or rituximab (Rtx), anti-CD16 mAb, MICA-His, ULBP2, anti-NKG2D mAb, or anti-NKp30 mAb, all with ICAM-1, for 5 min and fixed. F-actin was stained with Alexa Fluor 488–labeled phalloidin. Cells were scored according to their F-actin distribution; in some cells, F-actin accumulated in dense symmetrical rings (gray); in some, it accumulated asymmetrically in the leading edge (yellow), and in some cells, it was evenly distributed across the interface (green). **(B)** Cells were incubated on rituximab with ICAM-1 for 5, 10, or 30 min and fixed. F-actin was stained, and its distribution was assessed as in A. **(C–F)** NK cells were labeled with Calcein dye and put on rituximab (0, 1, or 10 µg/ml) or MICA (0, 0.25, or 2.50 µg/ml), both with ICAM-1. Brightfield, IRM, and fluorescence images were acquired for 45 min at 30-s intervals. Cell movements were quantitatively assessed using Cell Tracker, a MATLAB plugin. **(C)** Tracks of 20 individual cells from one donor on rituximab (0, 1, or 10 µg/ml) with ICAM-1. Axis lengths are ±300 µm. Tracks for ICAM-1 and 10 µg/ml rituximab are replotted from Fig. 6 F . **(D)** Tracks of 20 individual cells from one donor on MICA (0, 0.25, or 2.50 µg/ml) with ICAM-1. Axis lengths are ± 300 µm. Tracks for ICAM-1 and 2.5 µg/ml MICA are replotted from Fig. 6 F. **(E)** Mean NK cell speed from four different donors on ICAM-1 and rituximab (1 or 10 µg/ml; mean ± SEM). **(F)** Mean NK cell speed from four different donors on ICAM-1 and MICA (0.25 or 2.5 µg/ml; mean ± SEM). **(G–M)** NK cells were incubated on surfaces coated with rituximab or MICA, both with ICAM-1, in the presence of increasing concentrations of TAPI-0 as indicated. Unstimulated (US) cells were used as controls. After 1 h, cells were removed, stained for CD16, NKG2D, and cell viability, and assessed by flow cytometry. **(G and I)** Representative histograms of CD16 on unstimulated cells (shaded gray), cells activated on rituximab (red), cells activated on MICA (green), and cells treated with TAPI-0 in either condition (blue). Vertical line denotes $CD16^+$ cells. **(H and J)** gMFI of CD16 expression of $CD16^+$ cells normalized to unstimulated control cells upon activation on rituximab (H) or MICA (J). *n* = 3 independent experiments (mean ± SD). **(K)** gMFI of CD16 expression of $CD16^+$ cells normalized to unstimulated control cells. NK cells were stimulated on glass slides coated with ICAM-1 alone (–) or rituximab or MICA (both with ICAM-1) in the presence or absence of TAPI-0. **(L)** gMFI of NKG2D expression plotted for total cell population and normalized to unstimulated cells. **(M)** Cell viability was assessed using dead cell marker. Cell debris was gated out before assessment. **(K–M)** *n* = 7 independent experiments for US, rituximab, and MICA; *n* = 2 for ICAM-1 (mean ± SD). **(N)** Flow cytometry analysis of NK cell degranulation based on degranulation marker CD107a. NK cells were stimulated on glass slides coated with ICAM-1 alone (–), or rituximab or MICA (both with ICAM-1) and after 4 h, cells were removed and stained with Alexa fluor 647–labeled anti-CD107a mAb. Plotted values are CD107a gMFI normalized to cells stimulated on ICAM-1 (*n* = 6 symbols indicate different donors; mean ± SD). **(O)** NK cells were incubated in 96-well plates coated with ICAM-1, or rituximab or MICA (both with ICAM-1) for 18 h. IFN-γ production was measured from the supernatants by ELISA (*n* = 5 symbols indicate different donors; mean ± SD). *, $P < 0.05$; **, $P < 0.01$; ****, $P < 0.0001$ calculated by Student's *t* test.

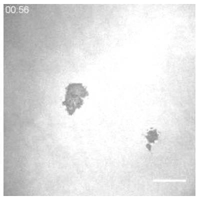

Video 1. **NK cell interactions with rituximab-coated surfaces.** IRM live-cell imaging of the contact interface between NK cells and glass slides coated with rituximab with ICAM-1. Images were acquired at a rate of one frame per second. Display rate of the video is 20 frames per second. Time is indicated as minutes:seconds. Bar, 20 µm.

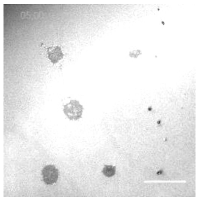

Video 2. **NK cell interactions with MICA-coated surfaces.** IRM live-cell imaging of the contact interface between NK cells and glass slides coated with MICA with ICAM-1. Images were acquired at rate of one frame per second. For the video, stacks were reduced 1:5. Display rate of the video is 20 frames per second. Time is indicated as minutes:seconds. Bar, 20 µm.

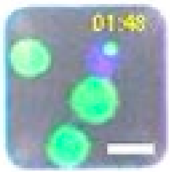

Video 3. **NK cell detaches from an opsonized target cell.** NK cells are labeled in blue, Daudi-rituximab cells are green, and dead cells are stained in red. Yellow indicates cells in an early stage of dying (losing green and obtaining red fluorescence). Representative video of one well (50 × 50 μm^2) showing an overlay of fluorescence and brightfield images at times indicated (hours:minutes). Display rate of the video is 10 frames per second. Bar, 20 µm.

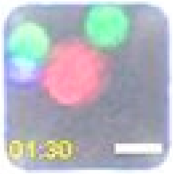

Video 4. **TAPI-0 prevents the detachment from an opsonized target cell.** NK cells are labeled in blue, Daudi-rituximab cells are in green, and dead cells are stained in red. Yellow indicates cells in an early stage of dying (losing green and obtaining red fluorescence). To inhibit ADAM17, TAPI-0 was added to the wells. Representative video of one well (50 × 50 μm^2) showing an overlay of fluorescence and brightfield images at times indicated (hours:minutes). Display rate of the video is 10 frames per second. Bar, 20 µm.
